# Hemicyanine-Based Near-Infrared Fluorescence Off–On Probes for Imaging Intracellular and In Vivo Nitroreductase Activity

**DOI:** 10.3390/ijms24076074

**Published:** 2023-03-23

**Authors:** Sun Hyeok Lee, Chul Soon Park, Kyung Kwan Lee, Tae-Hee Han, Hyun Seung Ban, Chang-Soo Lee

**Affiliations:** 1 Bionanotechnology Research Center, Korea Research Institute of Bioscience and Biotechnology (KRIBB), Daejeon 34141, Republic of Korea; tnsgur02@postech.ac.kr (S.H.L.); log71944@gmail.com (C.S.P.); lkk@kribb.re.kr (K.K.L.); 2 School of Interdisciplinary Bioscience and Bioengineering, Pohang University of Science and Technology (POSTECH), Pohang 37673, Republic of Korea; 3 Department of Bio-nanomaterials, Bio Campus of Korea Polytechnics, Nonsan 32943, Republic of Korea; 4 Department of Biomedical and Nanopharmaceutical Sciences, Graduate School, Kyung Hee University, Seoul 02447, Republic of Korea; 5 Biotherapeutics Translational Research Center, Korea Research Institute of Bioscience and Biotechnology (KRIBB), Daejeon 34141, Republic of Korea; gksxogml5739@kribb.re.kr; 6 Department of Bioscience, Korea Research Institute of Bioscience and Biotechnology School, University of Science & Technology (UST), Daejeon 34113, Republic of Korea; 7 Department of Biotechnology, Korea Research Institute of Bioscience and Biotechnology School, University of Science & Technology (UST), Daejeon 34113, Republic of Korea

**Keywords:** nitroreductase, fluorescent probes, near-infrared, bioimaging, mitochondria

## Abstract

Nitroreductase (NTR) has the ability to activate nitro group-containing prodrugs and decompose explosives; thus, the evaluation of NTR activity is specifically important in pharmaceutical and environmental areas. Numerous studies have verified effective fluorescent methods to detect and image NTR activity; however, near-infrared (NIR) fluorescence probes for biological applications are lacking. Thus, in this study, we synthesized novel NIR probes (**NIR-HCy-NO_2_ 1–3**) by introducing a nitro group to the hemicyanine skeleton to obtain fluorescence images of NTR activity. Additionally, this study was also designed to propose a different water solubility and investigate the catalytic efficiency of NTR. **NIR-HCy-NO_2_** inherently exhibited a low fluorescence background due to the interference of intramolecular charge transfer (ICT) by the nitro group. The conversion from the nitro to amine group by NTR induced a change in the absorbance spectra and lead to the intense enhancement of the fluorescence spectra. When assessing the catalytic efficiency and the limit of detection (LOD), including NTR activity imaging, it was demonstrated that **NIR-HCy-NO_2_ 1** was superior to the other two probes. Moreover, we found that **NIR-HCy-NO_2_ 1** reacted with type I mitochondrial NTR in live cell imaging. Conclusively, **NIR-HCy-NO_2_** demonstrated a great potential for application in various NTR-related fields, including NTR activity for cell imaging in vivo.

## 1. Introduction

Reductase is a kind of enzyme that chemically reduces a substrate. Nitroreductase (NTR) is a type of reductase and a type of flavoenzyme, which involves a nicotinamide adenine dinucleotide (phosphate) hydrate (NAD(P)H)-dependent reduction for nitro group-containing compounds, such as nitroaromatic and nitroheterocyclic molecules [1]. The determination of NTR activity is especially significant in vivo because most nitro group-containing compounds exhibit high cytotoxicity [2]. NTR has attracted great attention since it has started to be used as an activator of nitro group-containing prodrugs and a decomposer of explosives, such as trinitrotoluene (TNT) [3,4]. Therefore, the detection of NTR is highly significant in pharmaceutical and environmental areas. NTR is expressed in some bacteria and eukaryotic species, and it has been particularly utilized in therapeutic technologies for tumor-targeted delivery, such as cancer chemotherapy, also known as gene-directed enzyme prodrug therapy (GDEPT) [5,6,7,8,9].

NTR is classified into two different types: oxygen-insensitive type I NTR and oxygen-sensitive type II NTR. Type I NTR is mainly used as a nitroaromatic prodrug activator in GDEPT, and type II NTR is used for the selective imaging of hypoxic tumors because of its overexpression and the little interference that oxygen has under hypoxic conditions [10,11,12,13,14,15,16,17,18,19]. Further, previous studies on NTR activity fluorescence imaging have mostly focused on type II NTR [7,8,9] because fluorescence imaging for the activity of type I NTR is relatively insufficient. Previous reports have suggested the possibility of the mitochondrial existence of type I NTR based on the bacterial origin of mitochondria in fluorescence imaging [20,21,22]. As a prodrug activator, type I NTR can potentially present activity for nitroaromatic prodrugs when employing fluorescence imaging.

Functional fluorescent probes have the ability to identify and distinguish species of interest (SOI) in complex systems, such as intracellular or in vivo systems [23]. To utilize the advantages of functional fluorescent probes, various SOI have been applied to sensors and bioimaging, including for the detection and imaging of NTR. The probes for NTR detection are enzymatically activated by NTR, reducing the nitro group to the amine group, which induces a conversion from the electron-withdrawing group (EWG) to the electron-donating group (EDG) [2,24,25]. The enzymatic reduction induced by the activity of NTR results in a remarkable enhancement in the fluorescence spectra due to the intramolecular charge transfer (ICT) of the amine group, known as a strong EDG, utilizing the change in the functional group induced by NTR. However, most of the probes still suffer from the limitations of a high fluorescence background and a slow response, making the majority of probes useful only for the detection and imaging of NTR in cytoplasm. Moreover, the fluorescence probes proposed in previous studies were mostly viable in the visible range in order to assess the detection and imaging of NTR activities; however, fluorescence in the visible region is unfavorable for in vivo imaging due to the transmission and absorbance of biomolecules [26]. Therefore, near-infrared (NIR) probes are much more suitable for in vivo imaging due to their relatively low absorbance and good transmission in the NIR region.

**Figure 1:**
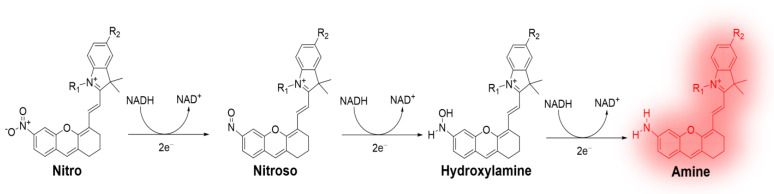
Reduction mechanism of **NIR-HCy-NO_2_** probe triggered by NTR.

In this study, we describe the novel NIR fluorescence probe **NIR-HCy-NO_2_** for the intracellular and in vivo imaging of NTR activity through the highly selective enzymatic reaction of NTR (Figure 1). We used a hemicyanine skeleton as a fluorogenic backbone, introducing a nitro group as a selective NTR-responsive moiety and fluorescence quencher. **NIR-HCy-NO_2_** derivatives (**NIR-HCy-NO_2_ 2** and **NIR-HCy-NO_2_ 3**) are also designed to increase water solubility, which was achieved by introducing a sulfonate (-SO_3_^−^) and quaternary ammonium group to the indolium part of **NIR-HCy-NO_2_** [27,28]. **NIR-HCy-NO_2_** showed a low fluorescence because of the interference of ICT by the nitro group and the reduction induced by NTR, which induced an enhancement in the fluorescence spectra due to the effect of ICT on the amine group. According to previous studies, indolium cation as a mitochondria tracker enables the verification of mitochondrial NTR [29]. This approach, which utilizes indolium cation in **NIR-HCy-NO_2_**, can be also applied to the mitochondrial imaging of NTR activity. Previously reported NTR probes have shown that the 4-nitrobenzene group, as an enzyme response moiety, should be introduced to the fluorophore backbone, and an additional elimination reaction is essential for the enhancement of the fluorescence [30,31]. In our newly synthesized **NIR-HCy-NO_2_** derivatives, the nitro group, the enzymatically reactive part, was directly conjugated to the NIR fluorophore backbone in order to achieve a fast response. The hemicyanine skeleton-based NIR probe has been previously applied to live zebrafish larvae for in vivo imaging. However, larval zebrafish have limitations in the in vivo imaging model due to their nonmammalian status and relatively thin skin, which is highly penetrable by light. Thus, fast responsive NIR-emitted NTR sensors should be used in NTR activity-related practical applications, such as in live cell and animal imaging.

Herein, the three derivatives of **NIR-HCy-NO_2_** are applied to intracellular and in vivo mouse imaging, respectively. It was hypothesized that **NIR-HCy-NO_2_** has high sensitivity and selectivity for NTR in the presence of NADH and that it would provide intracellular and in vivo NTR activity imaging.

## 2. Results

### 2.1. Design and Synthesis of NIR-HCy-NO_2_ 1–3

**Figure 2:**
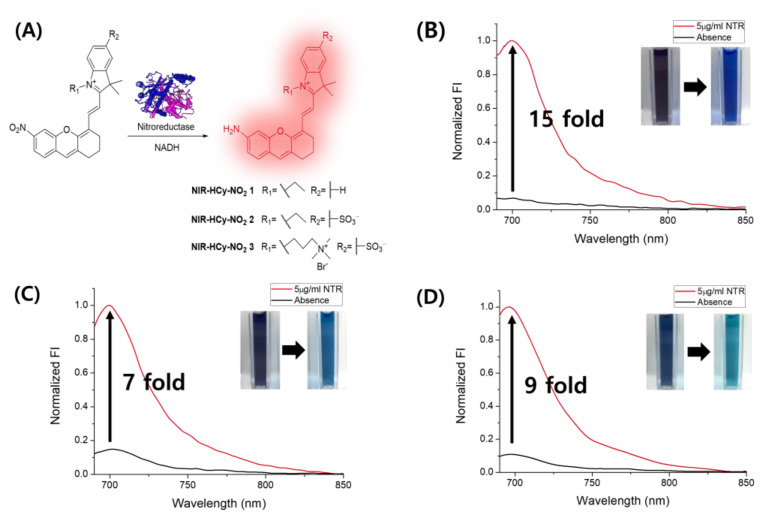
Nitroreductase (NTR)-mediated reduction and activation of **NIR-HCy-NO_2_** (**A**) and fluorescence enhancement of **NIR-HCy-NO_2_** in the presence of 5 µg/mL NTR and 50 µM NADH over 30 min (**B**–**D**). (**B**) **NIR-HCy-NO_2_ 1** in 1× PBS (pH 7.4, 20% (*v*/*v*) ACN), (**C**) **NIR-HCy-NO_2_ 2** in 1× PBS (pH 7.4, 5% (*v*/*v*) ACN), and (**D**) **NIR-HCy-NO_2_ 3** in 1× PBS (pH 7.4). The emission spectra were recorded using λ_ex_ = 672 nm.

To design and synthesize NIR fluorophore for NTR detection, the hemicyanine skeleton was chosen as a fluorescence unit because of its long wavelength (longer than λ = 650 nm) and ability to minimize autofluorescence and biological damage [32,33]. First, the bottom-up approach was reported to synthesize the NIR-emitted hemicyanine structure [34], and the structural flexibility was one of the attractive points for the use of the bottom-up approach. Previously, we developed novel alkaline phosphatase (ALP)-targeted NIR fluorescent probes (NIR-Phos-1 and NIR-Phos-2) using the same synthesis approach, and the hydroxyl group, which introduced the hemicyanine skeleton, was used as an NIR fluorophore for the ALP activity bioimaging [35]. In this study, we designed **NIR-HCy-NO_2_** derivatives, which are direct nitro group-modified hemicyanines. To achieve their synthesis, the nitro group, containing a tricyclic compound as a core intermediate, was combined with indolium derivatives which each have different functional groups (Scheme S1). **NIR-HCy-NO_2_ 2** and **NIR-Hcy-NO_2_ 3** had a better water solubility than **NIR-Hcy-NO_2_ 1** due to the introduction of sulfonate and quaternary ammonium to the indolium part in **NIR-Hcy-NO_2_**. As expected, the introduction of polar functional groups increased the water solubility of **NIR-Hcy-NO_2_**. **NIR-Hcy-NO_2_ 3** only reacted with NTR in PBS, and the **NIR-Hcy-NO_2_ 1** and **NIR-Hcy-NO_2_ 2** reactions were conducted in a cosolvent of PBS and ACN. However, **NIR-HCy-NO_2_ 1**, the most nonpolar probe, responded with the highest enhancement of fluorescence by NTR in the developed probes (Figure 2).

### 2.2. Optical Properties of NIR-HCy-NO_2_ 1–3

To confirm a change in the optical properties following the enzymatic reduction induced by NTR, we examined the differences in the absorbance spectra in the absence or presence of NTR. In the absorbance spectra, **NIR-HCy-NO_2_ 1–3** absorbed at λ_abs_ = 552, 594, and 600 nm in the absence of NTR, and the absorbance peak increased at λ_abs_ = 604, 662, and 658 nm in the presence of NTR and NADH (Figure S30) [36]. To explain the relationship between the change in the absorbance spectra and the enhancement in the fluorescence spectra, the reduction of **NIR-HCy-NO_2_ 1–3** by NTR was carried out under the same conditions, and the fluorescence signals of all **NIR-HCy-NO_2_ 1–3** were enhanced 15-, 7-, and 9-fold, respectively, compared to the absence of NTR (Figure 2B–D). Additionally, following the reduction induced by NTR, the color of the **NIR-HCy-NO_2_ 1** solution changed from violet to blue, **NIR-HCy-NO_2_ 2** changed from navy blue to blue, and **NIR-HCy-NO_2_ 3** changed from navy blue to emerald. These color changes in **NIR-HCy-NO_2_ 1–3** were caused by the bathochromic effect (i.e., redshift), as confirmed in the absorbance spectra. Additionally, the red light-absorbed fluorophores were generally observed as a blue-green color in the solution; a similar trend was observed in **NIR-HCy-NO_2_ 1–3**. High-resolution mass spectrometry (HR-MS) was used to show that the bathochromic effect was caused by the change from the nitro group to the amine group; **NIR-HCy-NO_2_ 1–3** were converted to hydroxylamine (**NIR-HCy-NHOH**) as the intermediate and were then reduced to amine (**NIR-HCy-NH_2_**) as the final product. The hydroxylamine intermediate *m*/*z* values of **NIR-HCy-NO_2_ 1–3** were calculated at 413.2224 ([**NIR-HCy-NHOH 1**]^+^), 515.1611 ([**NIR-HCy-NHOH 2** + Na]^+^), and 293.6209 ([**NIR-HCy-NHOH 3** + Na]^2+^) and observed to be 413.2231, 515.1613, and 293.6211, respectively. The amine product *m*/*z* values of **NIR-HCy-NO_2_ 1–3** were also calculated at 397.2274 ([**NIR-Hcy-NH_2_ 1**]^+^), 499.1662 ([**NIR-Hcy-NH_2_ 2** + Na]^+^), and 285.6235 ([**NIR-Hcy-NH_2_ 3** + Na]^2+^) and were found to be 397.2226, 499.1636, and 285.6252, respectively. Additionally, the azoxy form of **NIR-HCy-NO_2_ 1** was found at 403.2112 ([**NIR-HCy 1-N=NO-NIR-HCy 1**]^2+^ calculated *m*/*z* = 403.2092). **NIR-HCy-NO_2_ 1–3** were reduced, which was in line with the expected process (Figure 1) and confirmed in the abovementioned results (Figures S31–S33). In previous studies, **NIR-HCy-NHOH 1–3** were reduced via four-electron transfer, and **NIR-HCy-NH_2_ 1–3** were reduced through two-electron transfer from hydroxylamine intermediates. Specifically, the formation of azoxy compounds was induced by the reaction between hydroxylamine and nitroso intermediates to produce more stable azoxy compounds than the intermediate forms. This can be explained indirectly through the azoxy formation of **NIR-HCy-NO_2_ 1**, in which nitroso intermediates were produced during the NTR reduction process. Specifically, the bathochromic effect and fluorescence enhancement occurred when the nitro group (a strong EWG) was reduced to an amine group (a strong EDG) by NTR. As a result, the fluorescence emissions of **NIR-HCy-NO_2_ 1–3** themselves were very weak, which is consistent with their nonemissive characteristic; this was due to the quenching effect of the six-nitro substitution on the hemicyanine skeleton. However, reductive **NIR-HCy-NO_2_ 1–3** showed fluorescent properties due to the removal of ICT interference by the nitro group (Figure 2B–D) [37,38]. 

To confirm the exact excitation and emission wavelengths of reduced **NIR-HCy-NO_2_ 1–3** by NTR, the emission spectra were recorded using the same excitation wavelength (λ_ex_ = 672 nm) (Figure S34). All three probes emitted at longer wavelengths, in order from longest to shortest, of 3, 2, and 1 in the NIR region. Additionally, **NIR-HCy-NO_2_ 1–3** exhibited a Stokes shift of more than 20 nm, similar to the Stokes shift of other NIR dyes. Additionally, to cross-check the fluorescence-emitted form, fully reduced forms (**HCy-NH_2_ 1–3**) were obtained using the chemical reduction method (Figures S35–S37). The spectra of **HCy-NH_2_ 1–3** were almost the same as the NTR reduction result (Figures S34 and S38), and the conversion from a strong EWG to a strong EDG was quite important to emit fluorescence due to the ICT effect. Moreover, kinetically, the reduction reaction of **NIR-HCy-NO_2_ 1–3** samples by NTR was dependent on time and was saturated in less than 20 min (Figure S39). Specifically, the reductive reaction of **NIR-HCy-NO_2_ 1** and **NIR-HCy-NO_2_ 3** was saturated within 10 min (Figure S39A,C).

### 2.3. Selectivity Study

**Figure 3:**
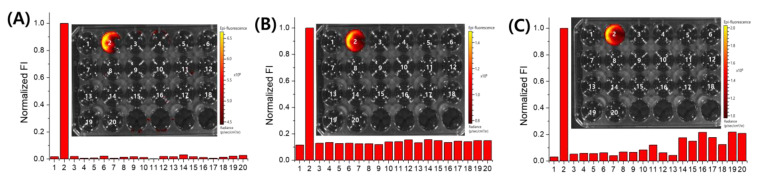
Fluorescence enhancement of **NIR-HCy-NO_2_** in the presence of alkali, alkaline earth metals, heavy metals, amino acids, and proteins. (**A**) **NIR-HCy-NO_2_ 1** in 1× PBS (pH 7.4, 20% (*v*/*v*) ACN), (**B**) **NIR-HCy-NO_2_ 2** in 1× PBS (pH 7.4, 5% (*v*/*v*) ACN), and (**C**) **NIR-HCy-NO_2_ 3** in 1× PBS (pH 7.4); reaction condition: 5 µM **NIR-HCy-NO_2_ 1–3** at 37 °C for 30 min; 1, **NIR-HCy-NO_2_**; 2, 5 µg/mL NTR + 50 µM NADH; 3, 50 mM NaCl; 4, 50 mM KCl; 5, 50 mM MgCl_2_; 6, 50 mM CaCl_2_; 7, 50 mM HgCl_2_; 8, 50 mM KBr; 9, 50 mM KI; 10, 1 mM L-cysteine; 11, 1 mM DL-homocysteine; 12, 1 mM L-phenylalanine; 13, 1 mM glycine; 14, 1 mg/mL BSA; 15, 1 U/mL GOx; 16, 1 U/mL thrombin; 17, 1 U/mL ALP; 18, 1 U/mL AchE; 19, 0.1 mg/mL lysozyme; 20, 0.1 mg/mL trypsin.

After confirming the reaction condition and optical properties of **NIR-HCy-NO_2_ 1–3**, including the reaction time, excitation, and emission wavelength, we evaluated the selectivity of **NIR-HCy-NO_2_ 1–3** for NTR over other various types of biological and chemical species. The fluorometric change in **NIR-HCy-NO_2_ 1–3** by NTR and other analytes was measured. Metal cations (Na^+^, K^+^, Mg^2+^, Ca^2+^, and Hg^2+^), halogen anions (Br^−^ and I^−^), amino acids (L-cysteine, DL-homocysteine, L-phenylalanine, and glycine), and proteins (BSA, ALP, GOx, thrombin, AchE, lysozyme, and trypsin) were used as the analytes. The fluorometric response of **NIR-HCy-NO_2_ 1–3** by NTR was much higher than the other analytes (Figure 3). Thus, **NIR-HCy-NO_2_ 1–3** reacted selectively with NTR, and, specifically, **NIR-HCy-NO_2_ 1** showed the highest fluorescence response compared to the others.

### 2.4. Quantitative Analysis

The quantitative analysis of **NIR-HCy-NO_2_ 1–3** was performed with various concentrations of NTR under the physiological condition (PBS (pH 7.4) in the presence of 50 μM of NADH at 37 °C). The fluorescence signals of **NIR-HCy-NO_2_ 1–3** increased the NTR concentration dependently. The trend line R-square (R^2^) values of **NIR-HCy-NO_2_ 1–3** were 0.9888, 0.9969, and 0.9964, respectively, and all three probes showed good linearity over the concentration range of 0.125–5 μg/mL of NTR (Figure S40). The detection limits of **NIR-HCy-NO_2_ 1–3** were 8, 114, and 181 ng/mL NTR, respectively. The sensitivity was the opposite trend to the probe polarity. Thus, the probe polarity was quite an important factor in deciding the detection limit.

### 2.5. Michaelis–Menten Kinetics

**Figure 4:**
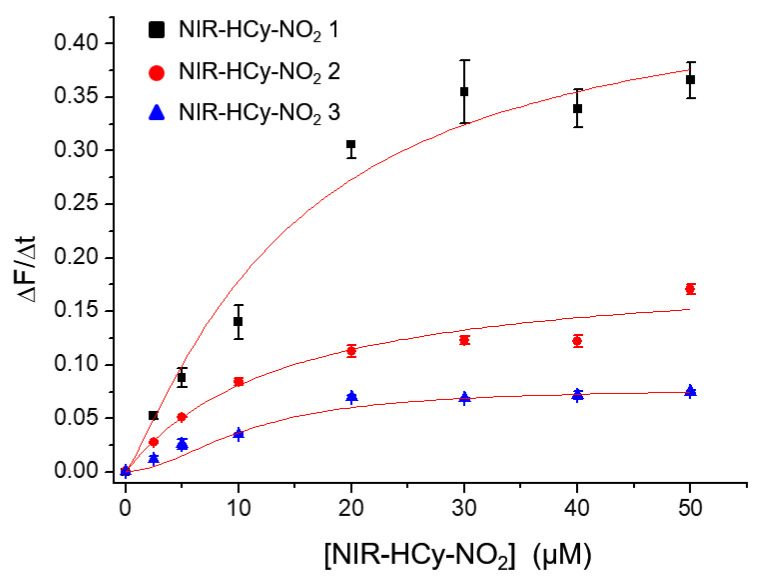
Michaelis–Menten curve of **NIR-HCy-NO_2_ 1–3** reacted with 5 µg/mL NTR and 50 µM NADH at 37 °C. **NIR-HCy-NO_2_ 1** reacted in 1× PBS (pH 7.4, 20% (*v*/*v*) ACN), **NIR-HCy-NO_2_ 2** in 1× PBS (pH 7.4, 5% (*v*/*v*) ACN), and **NIR-HCy-NO_2_ 3** in 1× PBS (pH 7.4).

To elucidate the enzyme–substrate interaction between NTR and **NIR-HCy-NO_2_ 1–3**, Michaelis–Menten kinetics were conducted. In Figure 4, the initial reaction rates of the enzymatic reduction were dependent on the concentration of **NIR-HCy-NO_2_ 1–3** (0–40 μM), and the reduction induced by NTR was saturated above 40 μM of **NIR-HCy-NO_2_ 1–3**. Consequently, the kinetic curves (Figure 4) followed the Michaelis—Menten equation. In the kinetics results, the Michaelis constant (K_m_) and the catalytic rate constant (k_cat_) of NTR for **NIR-HCy-NO_2_ 1–3** were obtained.

As shown in Table S1, the catalytic efficiency (k_cat_/K_m_) increased in the order of **NIR-HCy-NO_2_ 1–3**, and it was indicated that **NIR-HCy-NO_2_ 1** was reduced more effectively by NTR compared to **NIR-HCy-NO_2_ 2** and **NIR-HCy-NO_2_ 3**. Additionally, the K_m_ value of NTR for all three probes was lower than the kinetic values obtained in previous NTR probe studies under similar conditions. Thus, **NIR-HCy-NO_2_ 1–3** had a better affinity to NTR compared to the other probes reported in previous studies (Table S2). While **NIR-HCy-NO_2_ 1** was superior to nitrofurazone in terms of its catalytic efficiency, **NIR-HCy-NO_2_ 2** and **NIR-HCy-NO_2_ 3** were lower. The above results suggest that **NIR-HCy-NO_2_ 1** is a more suitable substrate for NTR kinetics analysis than the other two probes. It was also suggested that the charged function groups (sulfonate and quaternary ammonium) in indolium interrupt the reduction induced by NTR.

### 2.6. NTR Activity Imaging in Live Cells

We expanded the biological application of **NIR-HCy-NO_2_ 1–3** to NTR activity imaging in live cells. The A549 cell line was selected for confocal fluorescence images. First, the cytotoxicity tests of **NIR-HCy-NO_2_ 1–3** were performed to determine the probes’ nontoxic concentration level. All the probes were nontoxic at high concentrations (≥20 μM) for cell imaging after 1 h, suggesting that **NIR-HCy-NO_2_ 1–3** were biocompatible (Figure S41) for live cell NTR activity imaging.

**Figure 5:**
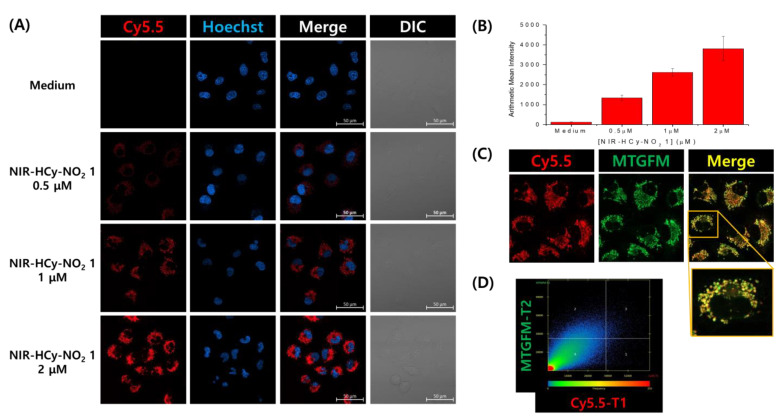
Confocal fluorescence images and arithmetic mean intensity graph of living A549 cells stained with various concentrations of **NIR-HCy-NO_2_ 1** (**A**,**B**). Colocalization of **NIR-HCy-NO_2_ 1** and MTGFM in living A549 cells (**C**,**D**). (**A**) Fluorescence and bright images of living A549 cells stained with various concentrations of **NIR-HCy-NO_2_ 1**. (**B**) The arithmetic mean intensity dependent on the treated concentration of **NIR-HCy-NO_2_ 1**. (**C**) Fluorescence images of **NIR-HCy-NO_2_ 1** and MTGFM and a merged image. (**D**) Intensity scatter plot of **NIR-HCy-NO_2_ 1** and MTGFM. The cell experiments were performed under normoxia conditions.

At a low concentration (<5 μM), **NIR-HCy-NO_2_ 1** induced sufficient fluorescence signals for imaging after 10 min (Figure 5A). However, the fluorescence signals of **NIR-HCy-NO_2_ 2** and **NIR-HCy-NO_2_ 3** were not detected at 40 μM after 30 min of treatment (Figure S42A). The arithmetic mean intensity of **NIR-HCy-NO_2_ 1** increased in a concentration-dependent manner; the arithmetic mean intensity of each concentration of **NIR-HCy-NO_2_ 1** increased 10.1-, 19.8-, and 28.8-fold, respectively, compared to the untreated group (Figure 5B). However, the arithmetic mean intensities of **NIR-HCy-NO_2_ 2** and **NIR-HCy-NO_2_ 3** increased up to about 6.2- and 2.7-fold, respectively (Figure S42B), which was too small a fold change considering their treatment concentrations. In the Michaelis–Menten kinetics results, the catalytic efficiencies of **NIR-HCy-NO_2_ 2** and **NIR-HCy-NO_2_ 3** were relatively lower than **NIR-HCy-NO_2_ 1**, and the trend was the same as live cell imaging (Figure 4, Figure 5 and Figure S42 and Table S2). Additionally, **NIR-HCy-NO_2_ 1** was localized in mitochondria, which was overlapped with a MitoTracker (Figure 5C). The scatter plot of the two channels (MitoTracker (MTGFM) and **NIR-HCy-NO_2_ 1** (Cy5.5)) showed a linear form and tendencies to synchronize with a Mender’s colocalization coefficient of 0.9748 (Figure 5D).

Interestingly, the **NIR-HCy-NO_2_ 1** signal was oxygen concentration-independent in the live cell (Figure S43). In a previous study, a Cy7-based fluorescence probe detected mitochondrial NTR in an A549 cell line under normoxia, and it was identified as type I NTR, which is oxygen-independent [20]. In this study, **NIR-HCy-NO_2_ 1** was also able to detect type I mitochondrial NTR under the same conditions and cell line. Moreover, the fluorescence signals of **NIR-HCy-NO_2_ 1** were similar under normoxia and hypoxia, and this suggested that **NIR-HCy-NO_2_ 1** was mainly reduced by type I mitochondrial NTR (Figure S43B). To verify the relationship between the signal enhancement and reductase, dicoumarol was pretreated as the reductase inhibitor, and the signal in the treated group decreased by up to 40% compared to the untreated group (Figure S44). As with previous results, **NIR-HCy-NO_2_ 1** is more suitable for NTR activity imaging than **NIR-HCy-NO_2_ 2** and **NIR-HCy-NO_2_ 3**.

### 2.7. In Vivo Imaging

**Figure 6:**
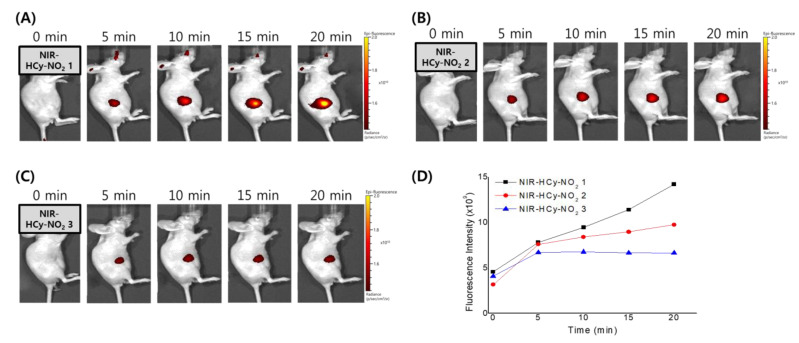
In vivo imaging of tumors with **NIR-HCy-NO_2_** in xenograft models. BALB/c nude mice bearing A549 tumors were injected intratumorally with 100 µL of PBS containing 20 µM of **NIR-HCy-NO_2_ 1** (**A**), **NIR-HCy-NO_2_ 2** (**B**), and **NIR-HCy-NO_2_ 3** (**C**). Fluorescence images were acquired at different time points after the injection of each probe (**D**).

We next applied **NIR-HCy-NO_2_** to in vivo fluorescence imaging in a xenograft model using an IVIS Spectrum system. Prior to imaging, we first determined the adequate concentration of each probe for injection. As shown in Figure S45, the fluorescent background signals from prereacted **NIR-HCy-NO_2_ 1–3** were not observed from 5–50 µM in PBS. Based on their cytotoxicity results, 20 µM was decided on for **NIR-HCy-NO_2_ 1–3**, considering a sufficiently strong fluorescence signal for in vivo imaging. Then, nude mice bearing A549 xenograft tumors were intratumorally injected with each probe and monitored in a treatment time–course manner. In Figure 6D, the fluorescence signal of all three **NIR-HCy-NO_2_** initially enhanced after the injection, and **NIR-HCy-NO_2_ 1** showed the strongest fluorescence signal among them. The fluorescence of **NIR-HCy-NO_2_ 1** was not saturated until 20 min post-injection, while that of **NIR-HCy-NO_2_ 2** and **NIR-HCy-NO_2_ 3** were saturated at 10 and 5 min, respectively. Thus, **NIR-HCy-NO_2_ 1** showed the best performance for in vivo tumor imaging among all **NIR-HCy-NO_2_** probes due to the strong signal and the continuous reaction with the reductase (Figure 6D).

## 3. Discussion

In summary, we succeeded in the design and synthesis of novel NIR off–on probes **NIR-HCy-NO_2_ 1–3** for NTR activity imaging. The water solubility of **NIR-HCy-NO_2_** derivatives was different depending on the functional group introduced to **NIR-HCy-NO_2_**, and all **NIR-HCy-NO_2_ 1–3** reacted selectively with NTR. The fluorescence intensity and absorbance spectra of **NIR-HCy-NO_2_ 1–3** changed due to the reduction reaction induced by NTR, and the LODs of **NIR-HCy-NO_2_ 1–3** were under 200 ng/mL of NTR. Among them, the LOD of **NIR-HCy-NO_2_ 1** was the lowest with 8 ng/mL of NTR, and the catalytic efficiency was the highest at 0.22 ± 0.03 μM^−1^·s^−1^. In intracellular NTR activity imaging, the performance of **NIR-HCy-NO_2_ 1** was overwhelmingly good, and the signal was reduced with a dicoumarol treatment known as the reductase inhibitor. Additionally, the **NIR-HCy-NO_2_ 1** response was oxygen-independent, and it was considered that **NIR-HCy-NO_2_ 1** should be used to detect and image type I mitochondrial NTR as the potential target in live cells. **NIR-HCy-NO_2_ 1** showed a strong fluorescence intensity and sustained reactivity in vivo. In conclusion, **NIR-HCy-NO_2_ 1** showed a good performance among the three derivatives, and similar trends were observed in the Michaelis–Menten kinetics and intracellular and in vivo NTR activity imaging. In further studies, **NIR-HCy-NO_2_** has the potential to be applied to various fields related to NTR, and it is able to be used for intracellular and in vivo NTR activity imaging.

## 4. Materials and Methods

### 4.1. Characterization of Optical Properties

The optical property measurements were performed in 1× phosphate buffered saline (PBS) (10 mM, pH 7.4) containing acetonitrile (ACN). **NIR-HCy-NO_2_ 1** was measured in 1× PBS (pH 7.4, 20% (*v*/*v*) ACN), **NIR-HCy-NO_2_ 2** was measured in 1× PBS (pH7.4, 5% (*v*/*v*) ACN), and **NIR-HCy-NO_2_ 3** was measured in 1× PBS (pH 7.4) without ACN, respectively. All of the optical analyses were carried out using 5 μM of **NIR-HCy-NO_2_ 1–3**, and NADH was added to all three probe solutions until the amount reached 50 μM. The mixture was incubated at 37 °C for 30 min. The UV-visible spectra were measured using a spectrophotometer (DU800, Beckman Coulter, Brea, CA, USA), and the fluorescence was measured using a fluorescence spectrometer (FS-2, SCINCO, Seoul, Republic of Korea) and an imaging reader (CYTATION5, BioTek, Winooski, VT, USA), respectively. The fluorescence spectra were recorded in the range from 685 to 850 nm, with λ_ex_ = 672 nm from a xenon lamp.

### 4.2. Mass Analysis of Reduced NIR-HCy-NO_2_ 1–3

To prepare the reaction mixture for mass analysis, 500 μM of **NIR-HCy-NO_2_ 1–3** were reduced by 1 μM of NTR with 500 μM of NADH in PBS (10 mM, pH 7.4) at room temperature for 3 min. To quench the enzymatic reaction, β-mercaptoethanol was added to the reaction mixture until the concentration was 2%. The product mass was measured using a high-resolution mass spectrometer (micrOTOF-QII, Bruker Daltonik, Bremen, Germany) in the electrospray ionization (ESI) mode.

### 4.3. Cell Culture

Non-small cell lung cancer adenocarcinoma A549 cell lines were obtained from the Bioevaluation Center at the Korea Research Institute of Bioscience and Biotechnology (KRIBB). The cells were cultured in Dulbecco’s modified Eagle’s medium (DMEM) supplemented with 10% (*v*/*v*) fetal bovine serum (FBS) and 1% (*v*/*v*) penicillin/streptomycin (P/S). The cells were cultured at 37 °C under 5% CO_2_.

### 4.4. Cytotoxicity Assay

The cytotoxicity assay was carried out using the methylene blue staining method (Methylene blue, Sigma-Aldrich, St. Louis, MO, USA). A549 cells were seeded into 96-well cell culture plates at 2 × 10^4^/well and preincubated in DMEM (10% (*v*/*v*) FBS and 1% (*v*/*v*) P/S). After the medium in the wells was removed, **NIR-HCy-NO_2_ 1–3** (100 μL/well) at concentrations of 0–40 μM were added to the wells of the treatment group, respectively. The cells were incubated for 1 h at 37 °C under 5% CO_2_ and then fixed for over 1 h by adding 10% formalin solution (50 μL/well). After each well attached to fixed cells was washed with PBS (pH 7.4), the fixed cells were stained with 2% methylene blue working solution (50% (*v*/*v*) methanol) for 1 h. The stained cells were washed strongly with distilled water and dried sufficiently at room temperature. The cells were lysed using 0.5% hydrogen chloride, and an imaging reader (CYTATION5, BioTek, Winooski, VT, USA) was used to measure the OD600 (absorbance value) of each well. Cell viability was calculated using the following formula: cell viability (%T) = A_t_/A_c_ × 100 (%), where A_t_ denotes the absorbance value of the treated group, and A_c_ denotes the absorbance value of the untreated group.

### 4.5. Confocal Fluorescence Imaging in Living Cells

A549 cells (4 × 10^4^/well) were plated on µ-Slide 4 Well (ibidi, Gräfelfing, Germany) and were allowed to adhere for 24 h. All staining procedures were carried out under the normoxia condition. The cells were incubated in serum-free DMEM at 37 °C with **NIR-HCy-NO_2_ 1** (0.5, 1, and 2 μM) for 10 min, or **NIR-HCy-NO_2_ 2** and **NIR-HCy-NO_2_ 3** (40 µM) for 30 min. Then, the cells were washed with DPBS (0.4 mL × 2 times) and were further incubated with 5 μg/mL of Hoechst 33,342 in a serum-free DMEM at 37 °C for 5 min. After washing with DPBS (0.4 mL × 3 times), fluorescence imaging was performed with an LSM 800 confocal fluorescence microscope (ZEISS, Jena, Germany) at a 40× water immersion objective lens. The fluorescence signal of the cells incubated with Hoechst 33,342 and **NIR-HCy-NO_2_ 1–3** was collected at 400–600 nm using a semiconductor laser at 405 nm as an excitation resource of Hoechst 33,342 and at 645–700 nm using a semiconductor laser at 640 nm as an excitation resource of **NIR-HCy-NO_2_ 1–3**, respectively.

For the comparison of the fluorescence images between normoxia and hypoxia, the hypoxic culture was carried out for 12 h at 37 °C under 5% CO_2_ and 2% O_2_. The staining procedure of **NIR-HCy-NO_2_ 1** and Hoechst 33,352 and the condition of confocal fluorescence imaging were performed in the same way as previously outlined.

A549 cells were used for the colocalization imaging, and the confocal imaging for colocalization was carried out in the same culture and using the **NIR-HCy-NO_2_ 1** staining condition with fluorescence confocal imaging. Then, the cells were treated with 0.5 μM of MitoTacker^®^ Green FM (MTGFM) for 30 min and were washed with DPBS (0.4 mL × 3 times). Fluorescence imaging was carried out in the same conditions outlined for the previous experiment; the fluorescence signal was collected at 400–650 nm using a semiconductor laser at 490 nm as an excitation resource of MTGFM.

### 4.6. Reductase Inhibition Test in Live Cells

Dicoumarol (Sigma-Aldrich) was used for reductase inhibition. A549 cells were pretreated for 4 h with dicoumarol (500 µM), and then **NIR-HCy-NO_2_ 1** (10 µM) was added for 20 min. The fluorescence intensity in A549 cells was measured using a Synergy H1 multimode plate reader (BioTek instruments, Winooski, VT, USA).

### 4.7. Fluorescence Imaging in Xenograft Mice

The in vivo imaging of the probes was determined using a xenograft mouse model. All animal experimental protocols were approved by the bioethics committee of the KRIBB. Six-week-old female nude mice were subcutaneously inoculated with A549 cells (5 × 10^6^ cells) in the right flank. After two weeks, the mice were anesthetized with 2% isoflurane, and **NIR-HCy-NO_2_ 1–3** diluted in 100 µL of 1× PBS were injected into the tumor. The in vivo imaging was analyzed using the IVIS Lumina II luminescence imaging system (Caliper Life Science, Alameda, CA, USA) and Living Image software (Caliper Life Science).

## Data Availability

Not applicable.

## Supplementary Materials

The following supporting information can be downloaded at: https://www.mdpi.com/article/10.3390/ijms24076074/s1. References [39,40] are cited in Supplementary Materials.
