# Supplementary material for: Hemicyanine-Based Near-Infrared Fluorescence Off–On Probes for Imaging Intracellular and In Vivo Nitroreductase Activity
Source: Int J Mol Sci. 2023 Mar 23;24(7):6074. doi: 10.3390/ijms24076074 (PMC10094042; doi:10.3390/ijms24076074)
Supplement: Supplementary file 1 [file ijms-24-06074-s001.zip › ijms-2259883-supplementary.pdf]

Supplementary information

Hemicyanine-based near-infrared fluorescence off-on probes for intracellular and in vivo nitroreductase activity

Sun Hyeok Lee[1,2,†], Chul Soon Park[1,3,†], Kyung Kwan Lee[1,4], Tae-Hee Han[5,6], Hyun Seung Ban[5,6,*] and Chang-Soo Lee[1,7,*]

1 Bionanotechnology Research Center, Korea Research Institute of Bioscience and Biotechnology (KRIBB), Daejeon 34141, Republic of Korea

2 School of Interdisciplinary Bioscience and Bioengineering, Pohang University of Science and Technology (POSTECH), Pohang 37673, Republic of Korea

3 Department of Bio-nanomaterials, Bio Campus of Korea Polytechnics, Nonsan, 32943, Republic of Korea

4 Department of Biomedical and Nanopharmaceutical Sciences, Graduate School, Kyung Hee University, Seoul 02447, Republic of Korea

5 Biotherapeutics Translational Research Center, Korea Research Institute of Bioscience and Biotechnology (KRIBB), Daejeon 34141, Republic of Korea

6 Department of Bioscience, KRIBB School, University of Science & Technology (UST), Daejeon 34113, Republic of Korea

7 Department of Biotechnology, KRIBB School, University of Science & Technology (UST), Daejeon 34113, Republic of Korea

* Correspondence: banhs@kribb.re.kr (H.S.B.); cslee@kribb.re.kr (C.-S.L.)

† These authors contributed equally to this work.

Experimental details

Materials and analysis equipment for synthesis

All used compounds and solvents were purchased from Cambridge Isotope Laboratories (Tewksbury, MA, USA), Sigma Aldrich (St. Louis, MO, USA), Tokyo Chemical Industry (Tokyo, Japan), and Samchun chemicals (Seoul, Republic of Korea). TLC Silica gel 60 F254 (Merck) was used for analytical TLC and silica gel 60 (0.040-0.063 mm) (Merck) was used for silica gel chromatography. The relative fluorescence quantum yield method was chosen, and rhodamine B was exploited as the quantum yield standard.[1] The quantum yield was calculated according to the equation (1). ^{1}H and ^{13}C NMR spectra were obtained from AVANCE 400 (Bruker, USA) at Korea Advanced Institute of Science and Technology (KAIST), UNITY INOVA 600NB (Varian Medical Systems, USA) at Daejeon Bio Venture Town, and 500 MHz AVANCE III HD (Bruker, USA) at Pohang University of Science and Technology (POSTECH). For LC-MS analysis, HPLC (Agilent, 1260 series) was used with DAD (diode array detector), single quadrupole mass (Agilent, 6100 series), and C18 column (agilent, 50 x 2.1mm, 1.8 μm, 80 Å). The HR-MS measurement (Bruker, micrOTOF-QII) was entrusted to KAIST Analysis Center for Research Advancement (KARA) in KAIST.

$$\Phi_{fl}=\Phi_{ref}(m/m_{ref})(n/n_{ref})^2 \qquad (1)$$

Where the ref means the reference fluorophore. Φ is the fluorescence quantum yield, m is the slope of the trend line in the scatter plot (y axis, area under curve of fluorescence, x axis, absorbance), and n is the refractive index.

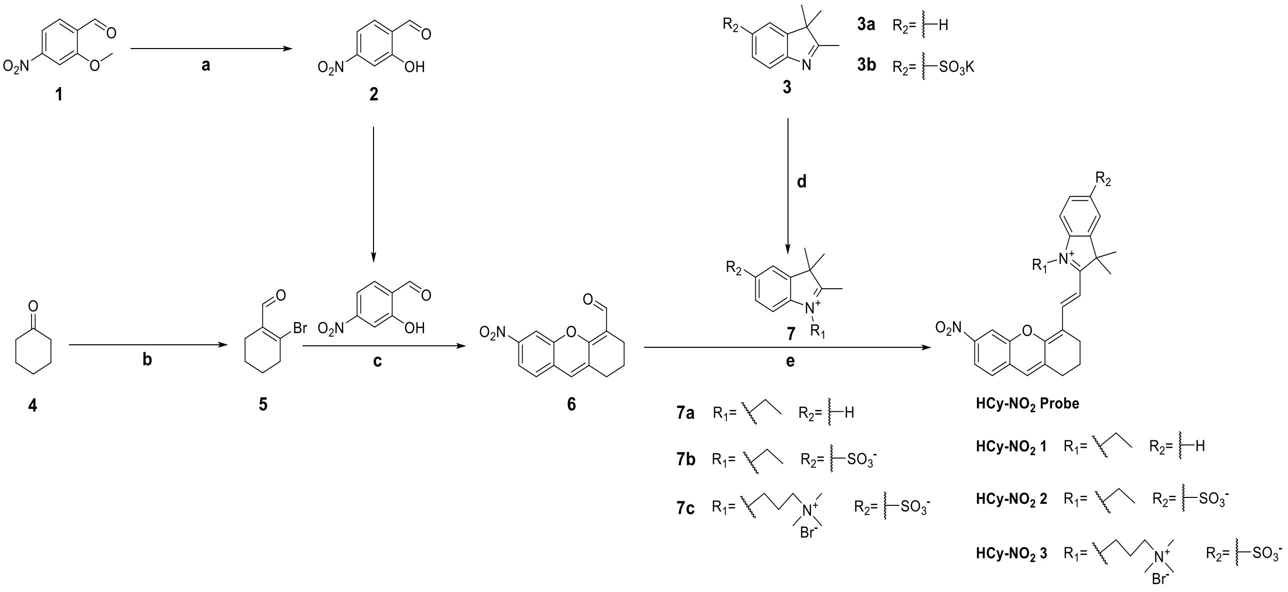

Scheme S1. Synthesis of NIR-HCy-NO_2 1-3. Conditions: (a) BBr_3, DCM, 0°C→RT; (b) PBr_3, DMF, $CHCl_3$ 0°C →RT; (c) $CsCO_3$, DMF, 25°C; (d) Acetonitrile, reflux; and (e) Acetic anhydride, 40 or 55°C.

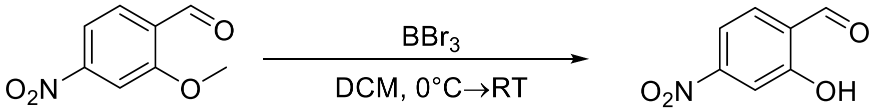

Figure S1. Synthesis of compound **2**.

Synthesis of 2-hydroxy-4-nitrobenzaldehyde (**2**): 2-methoxy-4-nitrobenzaldehyde (**1**) (1 g, 5.52 mmol) was dissolved in 56 mL CH_2Cl_2 and then purged with N_2 gas sufficiently. BBr_3 (1.97 mL, 20.42 mmol) was slowly added to the solution at 0˚C under N_2 gas. The color changed light yellow to red while adding BBr_3 to the solution. The reaction mixture was stirred for 19 hours at room temperature. The reaction mixture was poured into an ice bath. The aqueous layer was separated and extracted with ethyl acetate. The combined organic layer was washed with water, brine, dried over Na_2SO_4, filtered, and the residue was concentrated *in vacuo* to obtain orange colored solid (756.5 mg, yield 82%). ^{1}H NMR (400 MHz, $CDCl_3$): δ (ppm) 11.17 (s, 1H), 10.07 (s, 1H), 7.79-7.88 (m, 3H). ^{13}C NMR (150 MHz, DMSO-*d6*): δ (ppm) 189.83, 161.18, 151.93, 129.93, 127.17, 114.10, 112.51. HR MS [M-H]$^-$: m/z calcd 166.0146, found 166.0144.

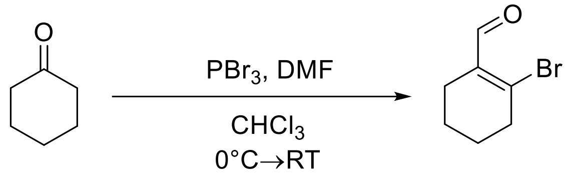

Figure S2. Synthesis of compound **5**.

Synthesis of 2-bromocyclohex-1-ene-1-carbaldehyde (**5**): PBr_3 (24.8 mL, 261 mmol) was slowly added to DMF (22.4 mL, 290 mmol) in 100 mL $CHCl_3$ at 0˚C. After 45 minutes, cyclohexanone (10 mL, 96.8 mmol) was added to the reaction mixture and stirred at room temperature for 16 hours. The reaction mixture was poured onto an ice bath and solid $NaHCO_3$ was slowly added until pH~7. Layers were separated and the aqueous layer was extracted with CH_2Cl_2. The organic layer was dried over Na_2SO_4, filtered and concentrated *in vacuo* to obtain orange oil, which was pure enough to be used directly in the next step. The product was to be rather volatile and unstable at room temperature, but could be stored for a few months under N_2 at -20˚C.

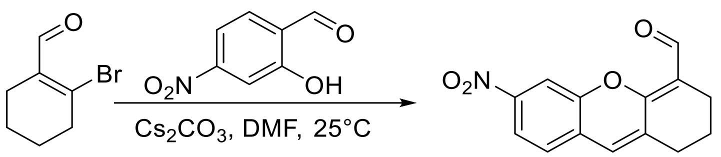

Figure S3. Synthesis of compound **6**.

Synthesis of 6-nitro-2,3-dihydro-1H-xanthene-4-carbaldehyde (**6**): Compound **5** (682 mg, 3.59 mmol) and cesium carbonate (2.9 g, 8.97 mmol) were added to compound **2** (500 mg, 2.99 mmol) in DMF (20 mL) with stirring under N_2 gas for 16 hours. The insoluble was filtered on celite, then the filtrate was concentrated *in vacuo*. The residue was dissolved in ethyl acetate and then extracted with water. The organic layer was washed with water and brine twice. The organic layer was dried over Na_2SO_4, and concentrated *in vacuo*. The residue was purified via flash column chromatography using CH_2Cl_2:MeOH (100:1) to obtain orange solid (330 mg, yield 35.7%). ^{1}H NMR (400 MHz, $CDCl_3$): δ (ppm) 10.38 (s, 1H), 7.93-7.95 (m, 2H), 7.29 (d, 1H, J = 2.16 Hz), 6.70

(s, 1H), 2.66 (t, 2H, *J* = 7.4 Hz), 2.48 (td, 2H, *J* = 6.08 Hz), 1.79 (qui, 2H, *J* = 6.16 Hz). ^{13}C NMR (150 MHz, DMSO-*d6*): δ (ppm) 188.10, 158.67, 151.44, 147.76, 134.15, 128.15, 127.32, 125.34, 119.43, 114.77, 110.98, 29.71, 21.41, 19.94. HR MS [M+Na]$^+$: m/z calcd 280.0580, found 280.0589.

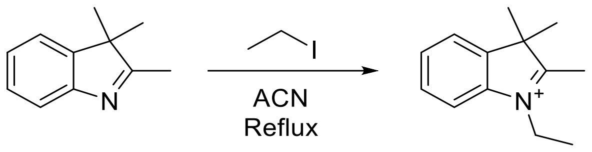

Figure S4. Synthesis of compound **7a**.

Synthesis of 1-ethyl-2,3,3-trimethyl-3H-indol-1-ium (**7a**): 2,3,3-trimethyl-3H-indole (**3a**) (1 g, 6.3 mmol) and iodoethane (1.52 mL, 18.9 mmol) were dissolved in 21 mL acetonitrile and the reaction mixture was refluxed for 36 hours under N_2 gas. The reaction mixture was concentrated *in vacuo*. The residue was dissolved in minimum amount of acetonitrile and then triturated in ethyl acetate. The purple solid was filtered and washed with ethyl acetate (1.8 g, yield 90%). ^{1}H NMR (400 MHz, DMSO-*d6*): δ (ppm) 7.92-7.94 (m, 1H), 7.79-7.82 (m, 1H), 7.57-7.60 (m, 2H), 4.48 (q, 2H, *J* = 7.28 Hz), 2.79 (s, 3H), 1.49 (s, 6H), 1.42 (t, 3H, *J* = 7.32 Hz). ^{13}C NMR (150 MHz, DMSO-*d6*): δ (ppm) 196.55, 142.40, 141.18, 129.83, 129.41, 124.00, 115.75, 54.57, 43.50, 22.33, 14.26, 13.13. HR MS [M]$^+$: m/z calcd 188.1434, found 188.1441.

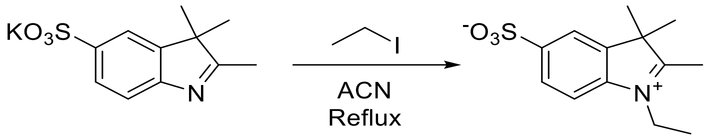

Figure S5. Synthesis of compound **7b**.

Synthesis of 1-ethyl-2,3,3-trimethyl-3H-indol-1-ium-5-sulfonate (**7b**): Potassium 2,3,3-trimethyl-3H-indole-5-sulfonate (**3b**) (300 mg, 1.08 mmol) and iodoethane (0.26 mL, 3.24 mmol) were dissolved in 3.6 mL acetonitrile and the reaction mixture was refluxed for 36 hours under N_2 gas. The purple solid was obtained via filter and washed with ethyl acetate and diethyl ether (213 mg, yield 74%). ^{1}H NMR (400 MHz, DMSO-*d6*): δ (ppm) 8.03 (sd, 1H, *J* = 1.28 Hz), 7.91 (d, 1H, *J* = 8.32 Hz), 7.83 (dd, 1H, *J* = 1.48, 8.4 Hz), 4.49 (q, 2H, *J* = 7.28 Hz), 2.81 (s, 3H), 1.54 (s, 6H), 1.44 (t, 3H, *J* = 7.32 Hz). ^{13}C NMR (150 MHz, DMSO-*d6*): δ (ppm) 197.33, 149.96, 142.06, 141.00, 126.81, 121.23, 115.18, 54.68, 43.60, 22.22, 14.28, 13.04. HR MS [M+K]$^+$: m/z calcd 306.0561, found 306.0582.

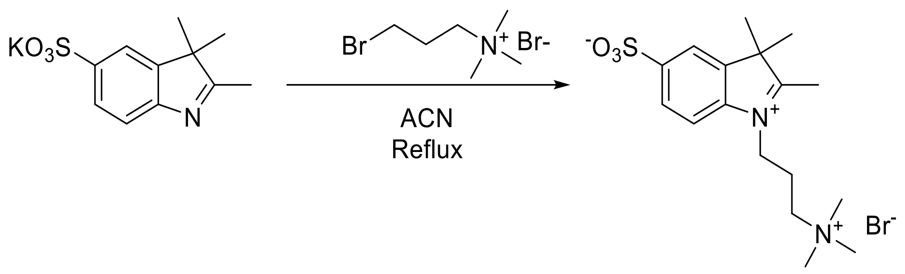

Figure S6. Synthesis of compound **7c**.

Synthesis of 2,3,3-trimethyl-1-(3-(trimethylammonio)propyl)-3H-indol-1-ium-5-sulfonate bromide (**7c**): 2,3,3-trimethyl-1-(3-(trimethylammonio)propyl)-3H-indol-1-ium-5-sulfonate bromide (**7c**) was synthesized following the procedure published by H. S. Choi *et al.* in (lit 81% yield [2]).

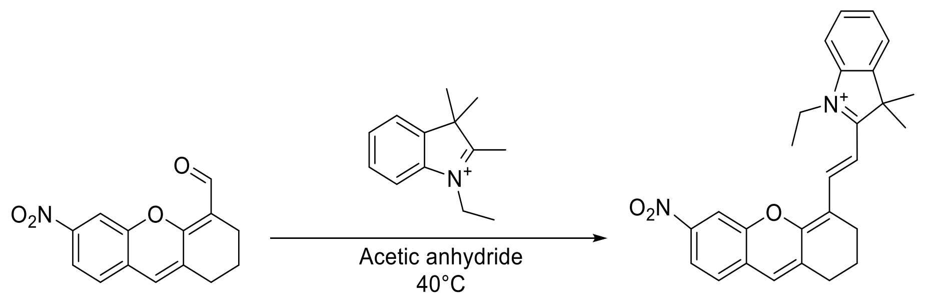

Figure S7. Synthesis of compound **NIR- HCy-NO$_2$ 1**.

Synthesis of (*E*)-1-ethyl-3,3-dimethyl-2-(2-(6-nitro-2,3-dihydro-1H-xanthen-4-yl)vinyl)-3H-indol-1-ium (**NIR-HCy-NO$_2$ 1**): Compound **6** (100 mg, 0.39 mmol) and compound **7a** (88 mg, 0.468 mmol) were stirred in Ac_2O (3.9 mL) at 40˚C under N_2 gas for 16 hours. The reaction mixture was extracted with ethyl acetate and washed with water and brine. Then the organic layer was dried over Na_2SO_4, filtered, and concentrated *in vacuo*. The residue was purified via flash column chromatography using CH_2Cl_2:MeOH (200:1, then 10:1) to obtain purple solid (24 mg, yield 11.1%). ^1H NMR (500 MHz, CD_3OD): δ (ppm) 8.83 (d, 1H, J = 15.4 Hz), 8.17 (s, 1H), 8.12 (dd, 1H, J = 0.6 Hz, 8.45 Hz), 7.78 (dd, 1H, J = 0.7 Hz, 7.15 Hz), 7.75 (d, 1H, J = 7.7 Hz), 7.57-7.64 (m, 3H), 7.26 (s, 1H), 6.82 (d, 1H, J = 15.4 Hz), 4.59 (q, 2H, J = 7.3 Hz), 2.84 (t, 2H, J = 5.65 Hz), 2.79 (t, 2H, J = 6.0 Hz), 2.01 (qui, 2H, J = 6.1 Hz), 1.80 (s, 6H), 1.57 (t, 3H, J = 7.25 Hz). ^{13}C NMR (125 MHz, CD_3OD): δ (ppm) 179.68, 157.84, 152.17, 148.40, 146.50, 143.11, 140.76, 134.36, 129.12, 128.48, 128.01, 127.81, 127.39, 122.67, 119.44, 115.71, 113.52, 110.57, 107.30, 51.61, 41.04, 29.21, 26.21, 23.51, 19.89, 11.93. HR MS $[M]^+$: m/z calcd 427.2016, found 427.2000.

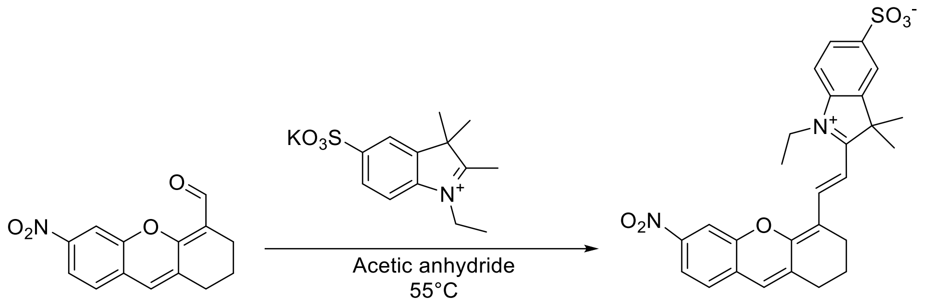

Figure S8. Synthesis of compound **NIR-HCy-NO_2 2**.

Synthesis of (E)-1-ethyl-3,3-dimethyl-2-(2-(6-nitro-2,3-dihydro-1H-xanthen-4-yl)vinyl)-3H-indol-1-ium-5-sulfonate (**NIR-HCy-NO_2 2**): Compound **6** (150 mg, 0.58 mmol) and compound **7b** (124 mg, 0.464 mmol) were dissolved in Ac_2O (5.8 mL) and then the reaction mixture was stirred at 55°C for 24 hours. Ethyl acetate was added to the reaction mixture to obtain bluish violet solid and the solid was washed with ethyl acetate. The solid was more purified via prep-HPLC to obtain bluish violet solid (31 mg, yield 10.6%). ^{1}H NMR (500 MHz, DMSO-*d6*): δ (ppm) 8.62 (d, 1H, *J* = 15.4 Hz), 8.33 (sd, 1H, *J* = 2.0 Hz), 8.11 (dd, 1H, *J* = 2.2 Hz, 8.45 Hz), 7.99 (sd, 1H, *J* = 1.3 Hz), 7.83 (dd, 1H, *J* = 1.5 Hz, 8.3 Hz), 7.78 (d, 1H, *J* = 8.35 Hz), 7.72 (d, 1H, *J* = 8.5 Hz), 7.39 (s, 1H), 6.81 (d, 1H, *J* = 15.5 Hz), 4.57 (q, 2H, *J* = 6.95 Hz), 2.76 (t, 2H, *J* = 5.55 Hz), 2.73 (t, 2H, *J* = 5.9 Hz), 1.82-1.86 (m, 8H), 1.43 (t, 3H, *J* = 7.2 Hz). ^{13}C NMR (125 MHz, DMSO-*d6*): δ (ppm) 179.93, 157.42, 152.22, 149.116, 148.23, 145.96, 143.16, 141.12, 134.78, 128.52, 128.45, 127.86, 127.06, 120.60, 120.34, 116.15, 114.01, 111.82, 108.83, 51.85, 41.73, 29.48, 27.14, 24.06, 20.04, 13.51. HR MS $[M+K]^+$: m/z calcd 545.1143, found 545.1142.

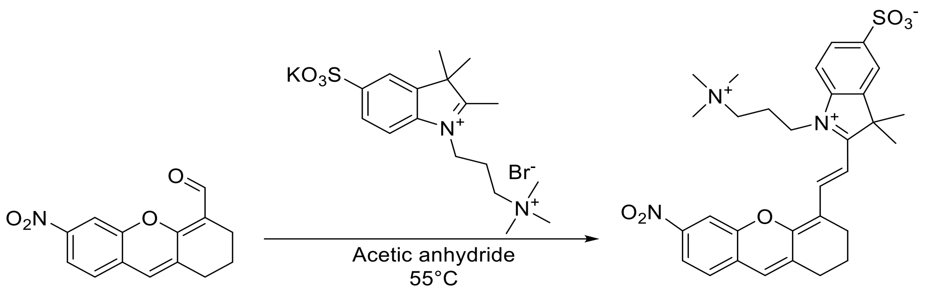

Figure S9. Synthesis of compound **NIR-HCy-NO_2 3**.

Synthesis of (E)-1-ethyl-3,3-dimethyl-2-(2-(6-nitro-2,3-dihydro-1H-xanthen-4-yl)vinyl)-3H-indol-1-ium-5-sulfonate (**NIR-HCy-NO_2 3**): Compound **6** (80 mg, 0.18 mmol) and compound **7c** (68 mg, 0.162 mmol) were dissolved in Ac_2O (1.8 mL) and then the reaction mixture was stirred at 55°C for 24 hours. Ethyl acetate was added to the reaction mixture to obtain bluish violet solid and the solid was washed with ethyl acetate. The solid was more purified via prep-HPLC to obtain navy solid (18 mg, yield 15.4%). ^{1}H NMR (500 MHz, CD_3OD): δ (ppm) 8.92 (d, 1H, *J* = 15.05 Hz), 8.26 (sd, 1H, *J* = 1.6 Hz), 8.16 (dd, 1H, *J* = 2.1 Hz, 8.45 Hz), 8.09 (s, 1H), 7.77-7.82 (m, 2H), 7.71 (d, 1H, *J* = 8.55 Hz), 7.40 (s, 1H), 6.83 (d, 1H, *J* = 15.1 Hz), 4.59 (t, 2H, *J* = 7.75 Hz), 3.71-3.74 (m, 2H), 3.26 (s, 9H), 2.82-2.88 (m, 4H), 2.45-2.51 (m, 2H), 2.01 (qui, 2H, *J* = 6.5 Hz), 1.91 (s, 6H). ^{13}C NMR (125

MHz, CD_3OD): δ (ppm) 181.17, 159.68, 152.23, 148.63, 148.11, 145.36, 142.76, 142.13, 134.31, 129.86, 128.12, 127.24, 127.07, 120.42, 119.73, 116.60 113.16, 110.94, 106.86, 62.77, 52.44, 51.65, 42.32, 29.17, 26.54, 23.59, 21.43, 19.87. HR MS $[M]^+$: m/z calcd 578.2319, found 578.2377.

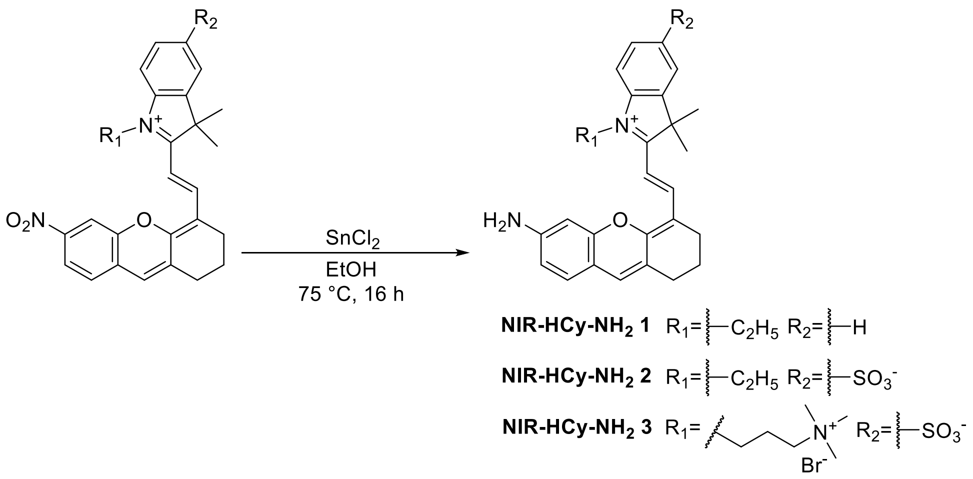

Figure S10. Chemical reduction of compound **NIR-HCy-NO2 1-3**.

Chemical reduction of NIR-HCy-NO2 1-3: NIR-HCy-NO2 1-3 (1 μmol) were dissolved in 270 μl EtOH and $SnCl_2$ (10 μmol) in EtOH was added. The reaction mixture was stirred at 75 °C for 16 h and the volatile was removed under reduced pressure to obtain **NIR-HCy-NH2 1-3**. **NIR-HCy-NH2 1** characterization: yield= 53.0%, ^{1}H NMR (500 MHz, CD_3OD): δ (ppm) 8.68 (d, 1H, J = 14.2 Hz), 7.56-7.58 (m, 2H), 7.49 (td, 1H, J = 1.1 Hz, 8.0 Hz), 7.39 (d, 2H, J = 8.5 Hz), 7.35 (t, 1H, J = 7.4 Hz), 6.79 (d, 1H, J = 8.3 Hz), 6.74 (sd, 1H, J = 1.55 Hz), 6.29 (d, 1H, J = 14.15 Hz), 4.28 (q, 2H, J = 6.85 Hz), 2.80 (br, 2H), 2.73 (br, 2H), 1.98 (qui, 2H, J = 6.3 Hz), 1.80 (s, 6H), 1.46 (t, 3H, J = 7.25 Hz). ^{13}C NMR (125 MHz, CD_3OD): δ (ppm) 173.71, 163.45, 156.23, 154.95, 142.75, 141.66, 141.21, 138.37, 129.54, 128.58, 125.34, 123.20, 122.14, 114.51, 114.35, 113.68, 110.78, 99.29, 97.59, 49.43, 38.96, 28.23, 27.21, 23.77, 20.53, 10.97. ESI-MS $[M]^+$: *m/z* calcd 397.2, found 397.4. **NIR-HCy-NH2 2** characterization: yield= 43.0%, ^{1}H NMR (500 MHz, DMSO-d6): δ (ppm) 8.44 (d, 1H, J = 13.85 Hz), 7.83 (sd, 1H, J = 1.25 Hz), 7.68-7.71 (m, 2H), 7.44 (d, 1H, J = 8.6 Hz), 7.41 (d, 1H, J = 8.35 Hz), 6.75 (dd, 1H, J = 1.85 Hz, 8.5 Hz), 6.74 (s, 1H), 6.26 (d, 1H, J = 13.8 Hz), 4.27 (q, 2H, J = 6.75 Hz), 2.72 (t, 2H, J = 5.9 Hz), 2.69 (t, 2H, J = 5.0 Hz), 1.84 (qui, 2H, J = 5.25 Hz), 1.71 (s, 6H), 1.32 (t, 3H, J = 7.1 Hz). ^{13}C NMR (125 MHz, DMSO-d6): δ (ppm) 173.20, 163.06, 156.28, 155.71, 146.19, 142.01, 141.70, 140.78, 139.39, 130.39, 126.75, 122.91, 120.38, 115.22, 114.83, 113.46, 110.81, 100.31, 97.71, 49.49, 49.06, 28.40, 28.20, 24.26, 20.69, 12.49. ESI-MS $[M+H]^+$: *m/z* calcd 477.2, found 477.2. **NIR-HCy-NH2 3** characterization: yield= 78.7%, ^{1}H NMR (500 MHz, CD_3OD): δ (ppm) 8.62 (d, 1H, J = 13.7 Hz), 7.92 (sd, 1H, J = 1.55 Hz), 7.84-7.86 (m, 2H), 7.53 (d, 1H, J = 8.7 Hz), 7.34 (d, 1H, J = 8.3 Hz), 6.91 (dd, 1H, J = 2.05 Hz, 8.7 Hz), 6.84 (sd, 1H, J = 1.4 Hz), 6.21 (d, 1H, J = 13.75 Hz), 4.23 (t, 2H, J = 7.5 Hz), 3.58-3.62 (m, 2H), 3.21 (s, 9H), 2.87 (t, 2H, J = 5.9 Hz), 2.81 (t, 2H, J = 6.0 Hz), 2.37 (qui, 2H, J = 8.05 Hz), 2.01 (qui, 2H, J = 5.9 Hz), 1.84 (s, 6H). ^{13}C NMR (125 MHz, CD_3OD): δ (ppm) 171.26, 164.66, 157.26, 156.93, 143.55, 142.32, 141.45, 140.39, 140.26, 130.41, 126.75, 122.83, 120.08, 116.68, 114.90, 109.13, 98.00, 96.87, 63.26, 52.32, 48.56, 40.05, 27.92, 27.59, 23.89, 20.60, 20.54. ESI-

MS [M]$^+$: *m/z* calcd 548.3, found 548.4; [M+H]$^{2+}$: *m/z* calcd 274.6, found 274.8. The HPLC chromatogram and ESI-MS spectrum of **NIR-HCy-NH$_2$ 1-3** are in Fig. S35-37.

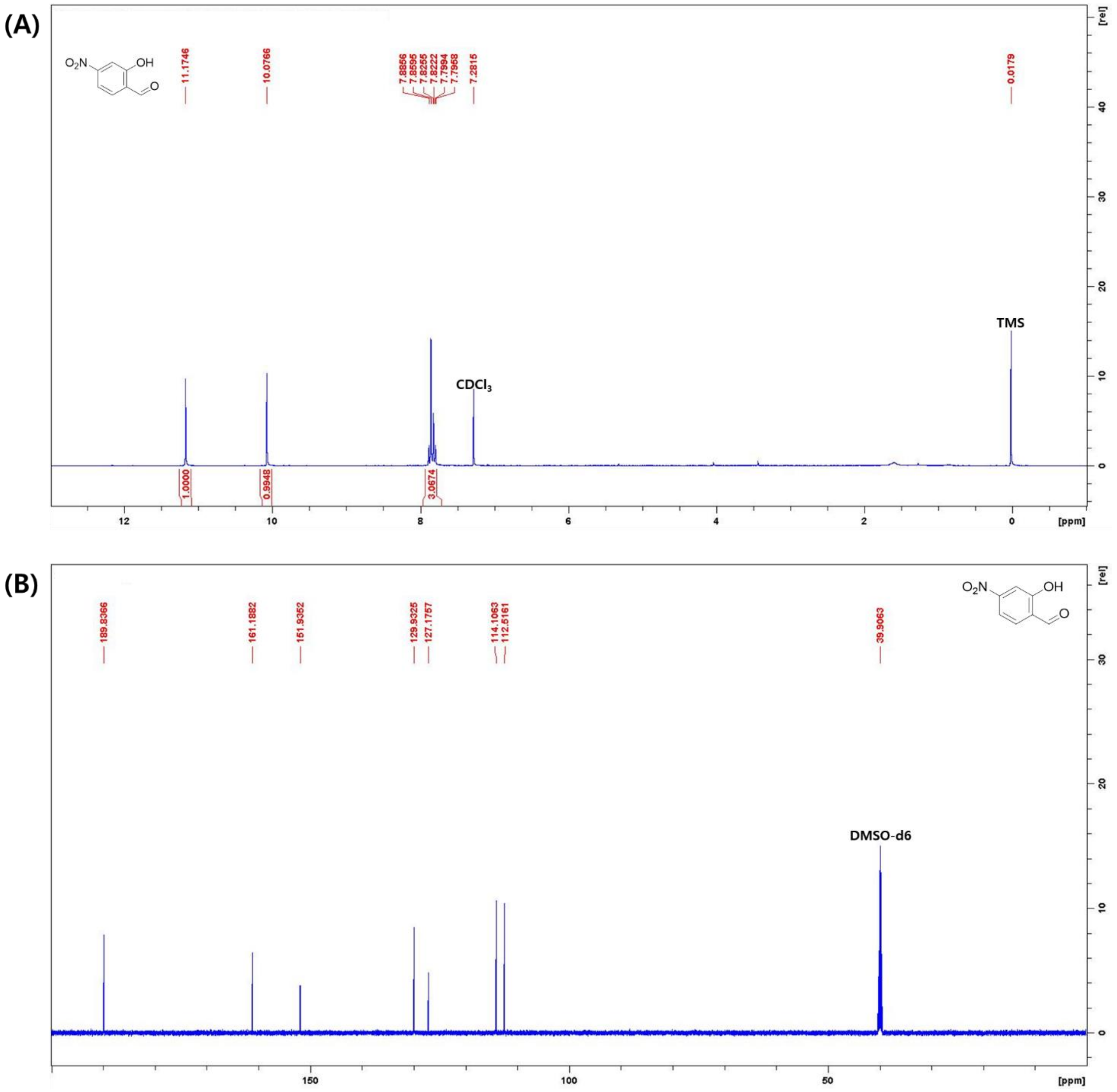

Figure S11. ^1H (A) and ^{13}C (B) NMR spectrum of compound **2**.

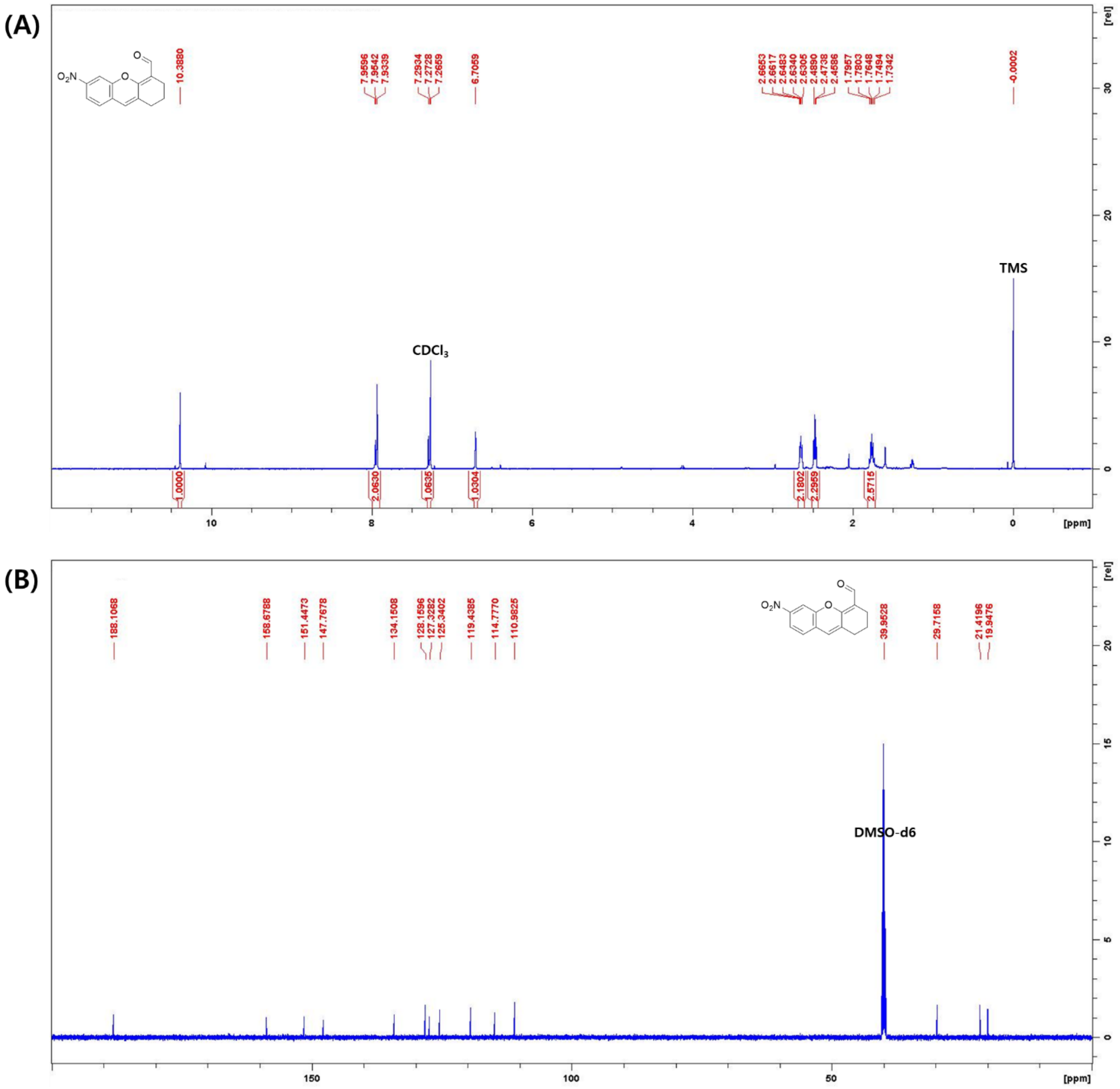

Figure S12. ^{1}H (A) and ^{13}C (B) NMR spectrum of compound **6**.

(A)

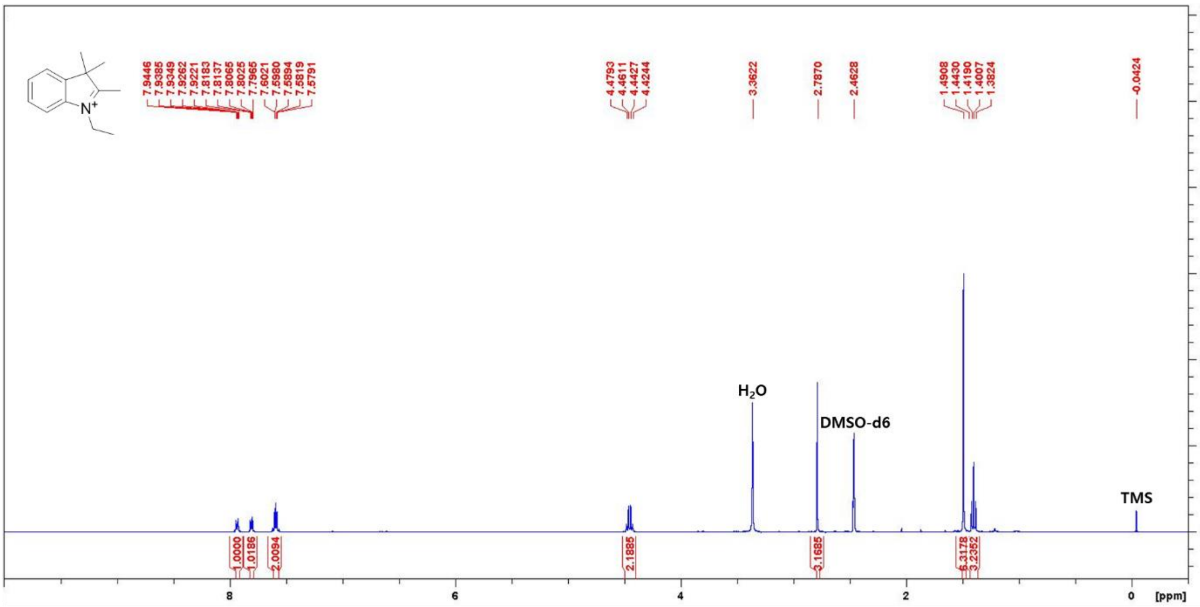

(B)

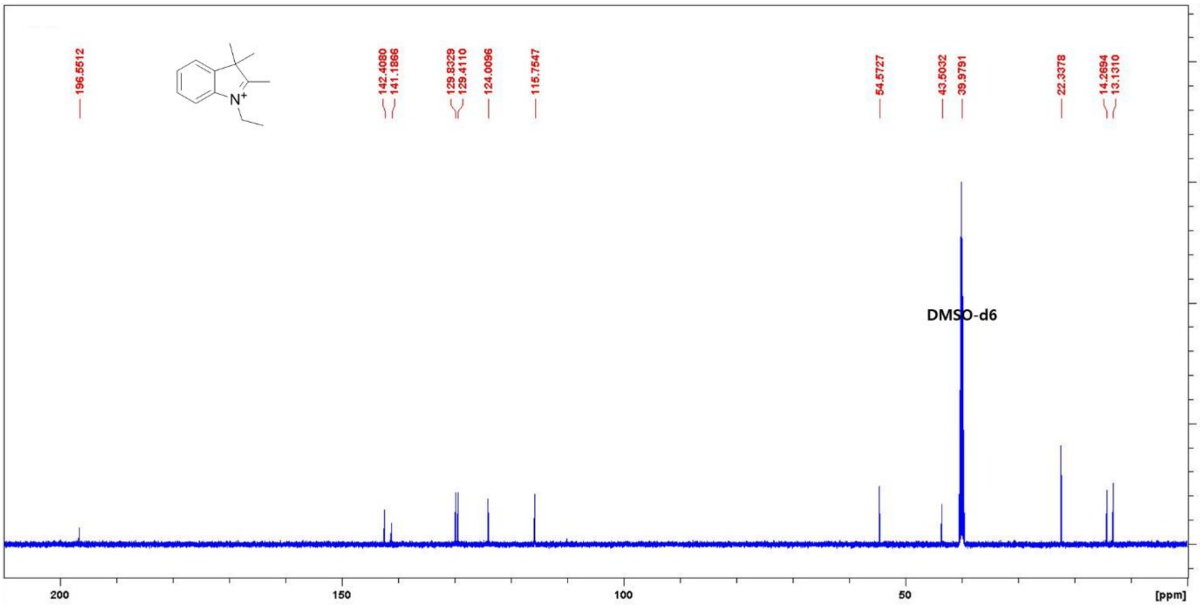

Figure S13. ^{1}H (A) and ^{13}C (B) NMR spectrum of compound **7a**.

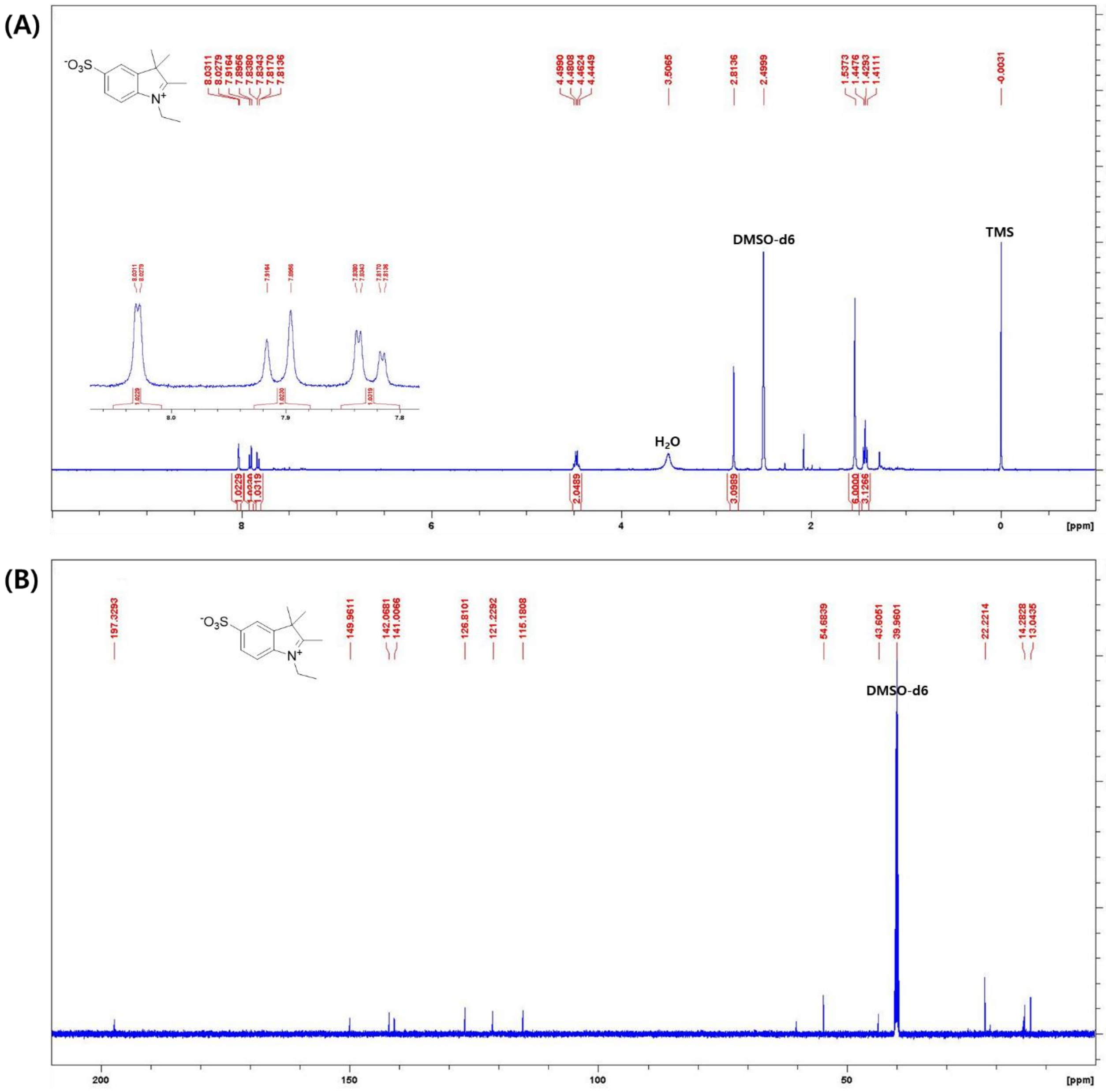

Figure S14. ^{1}H (A) and ^{13}C (B) NMR spectrum of compound **7b**.

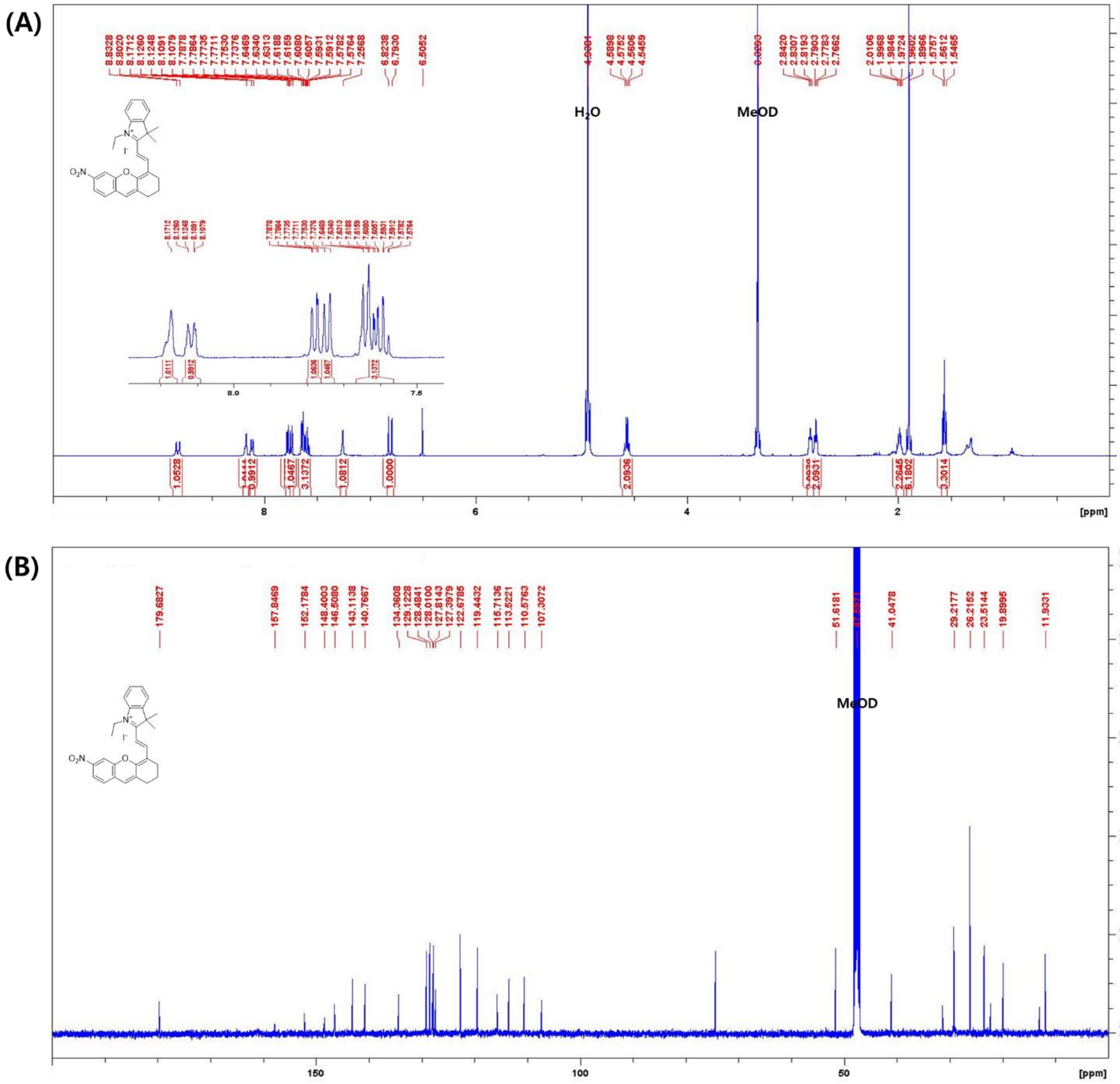

Figure S15. ^{1}H (A) and ^{13}C (B) NMR spectrum of compound **NIR-HCy-NO₂ 1**.

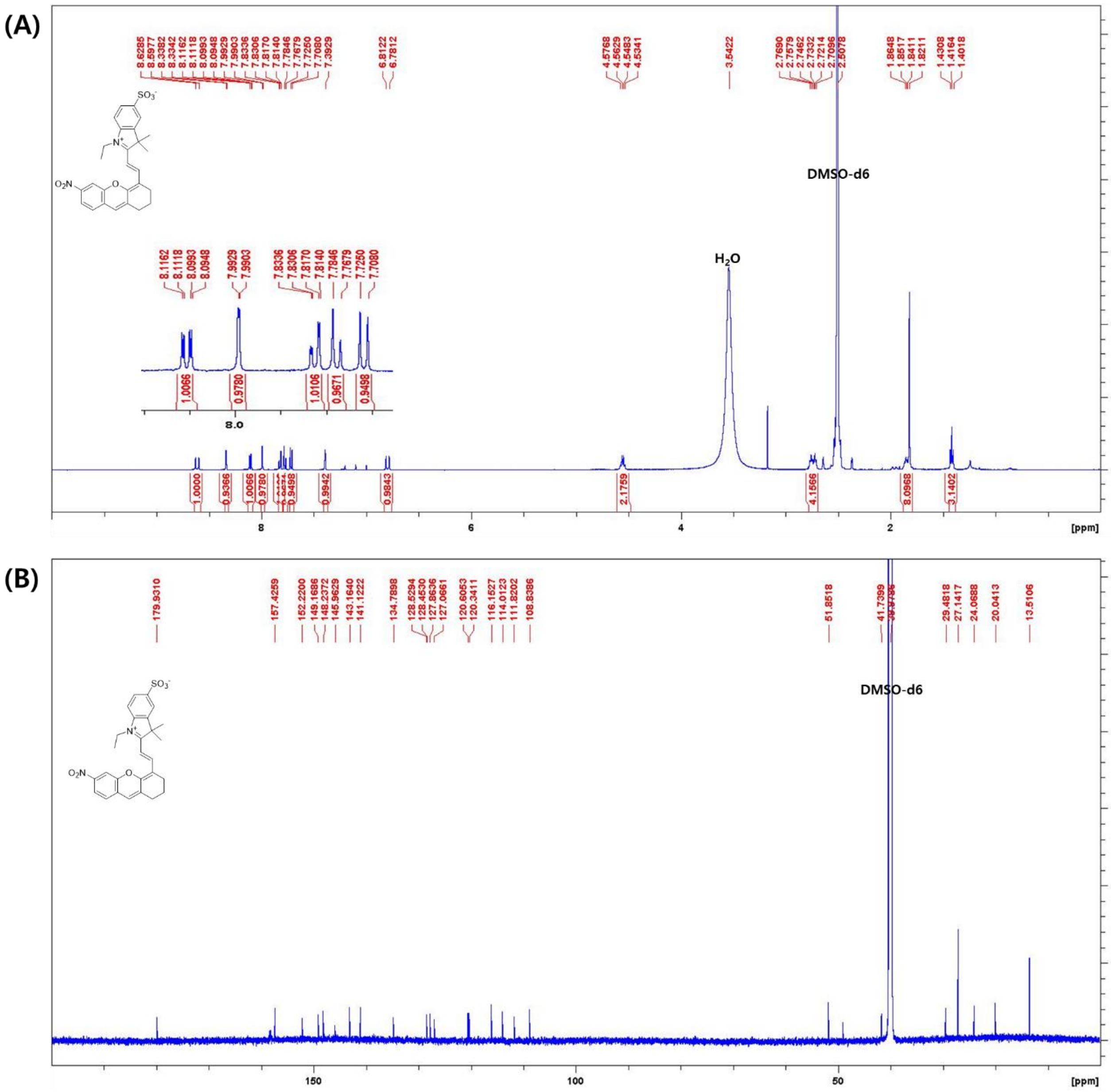

Figure S16. ^{1}H (A) and ^{13}C (B) NMR spectrum of compound **NIR-HCy-NO$_2$ 2**.

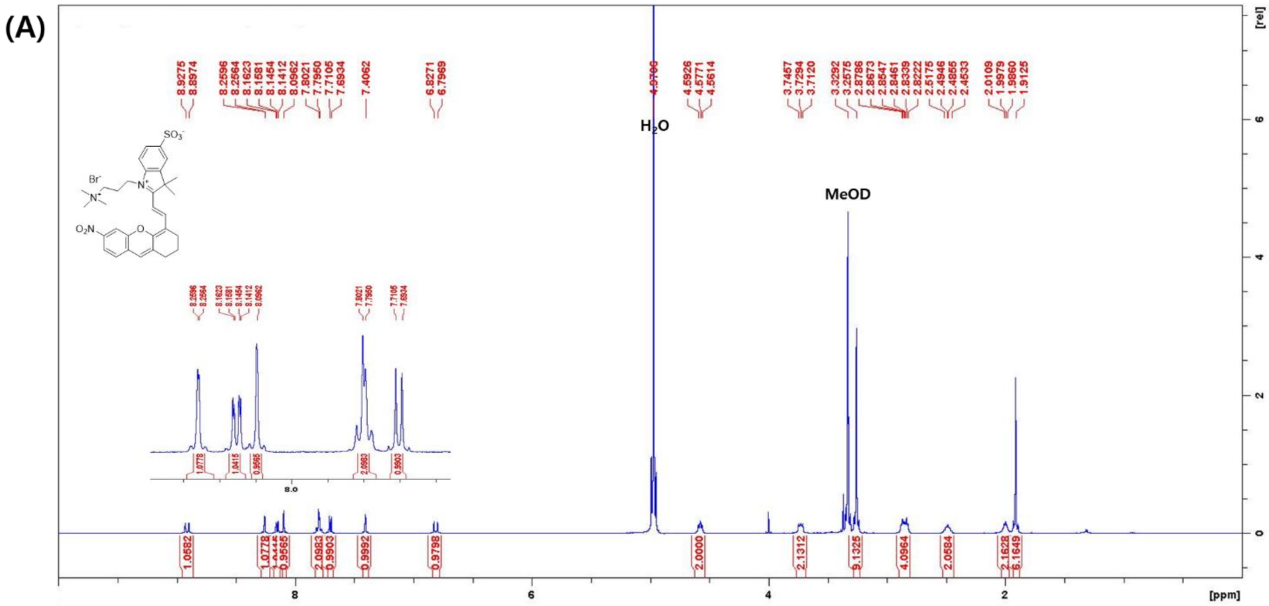

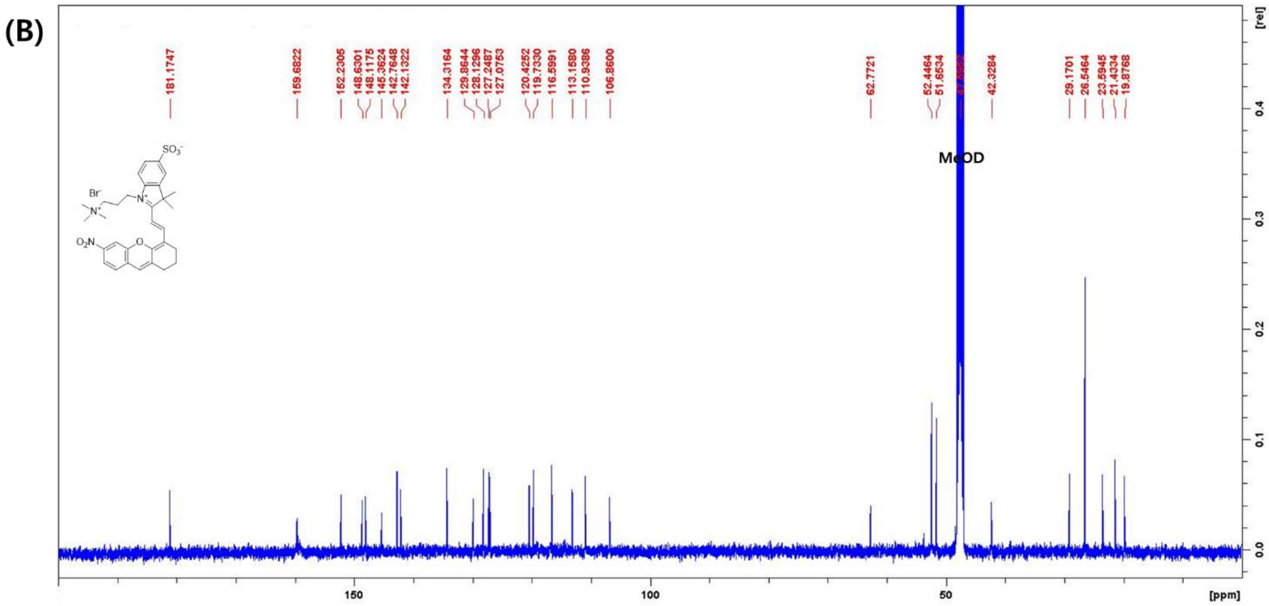

Figure S17. ^{1}H (A) and ^{13}C (B) NMR spectrum of compound **NIR-HCy-NO$_2$ 3**.

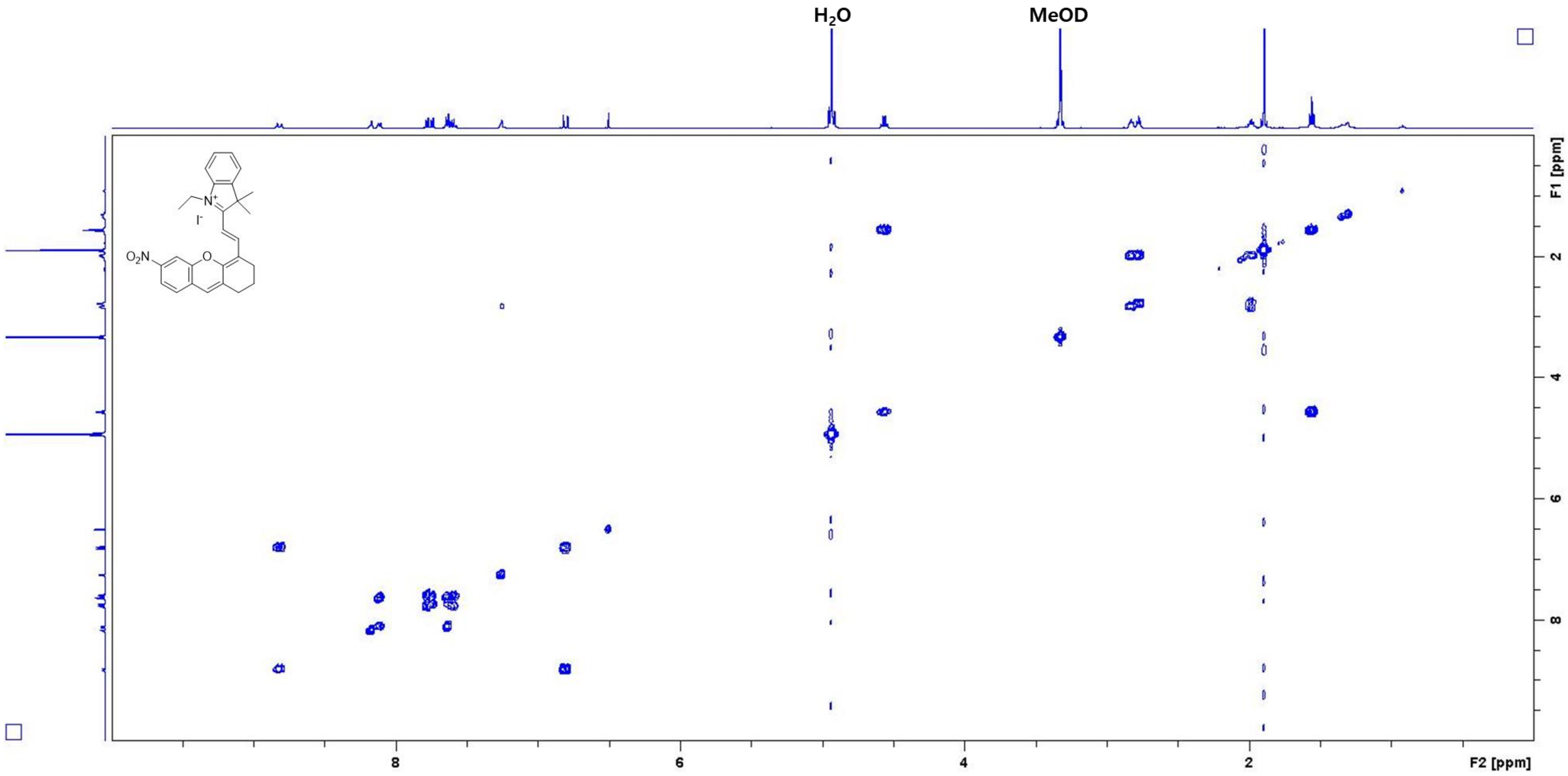

Figure S18. COSY spectrum of compound **NIR-HCy-NO₂ 1** (500 MHz).

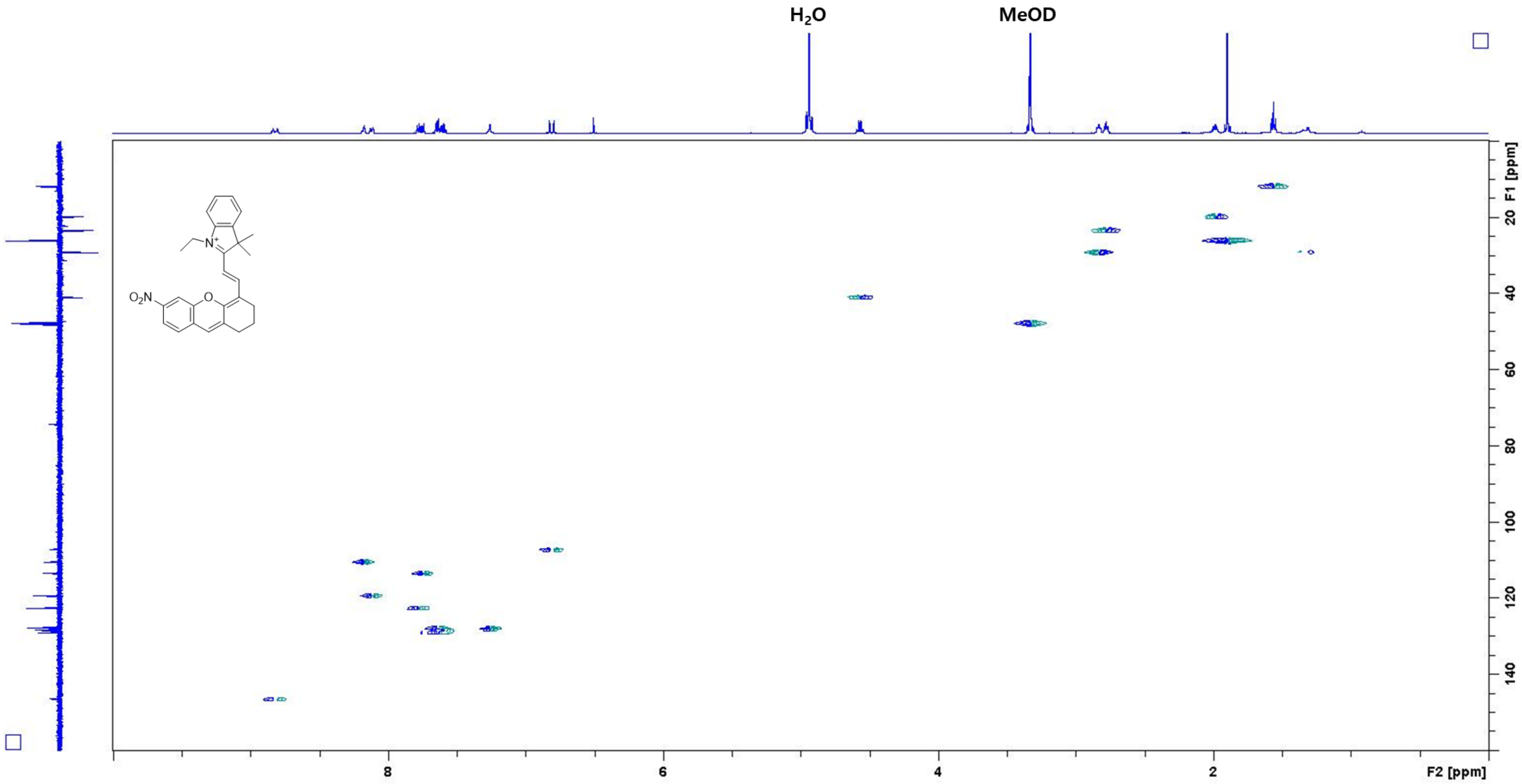

Figure S19. HSQC spectrum of compound **NIR-HCy-NO₂ 1** (500 MHz).

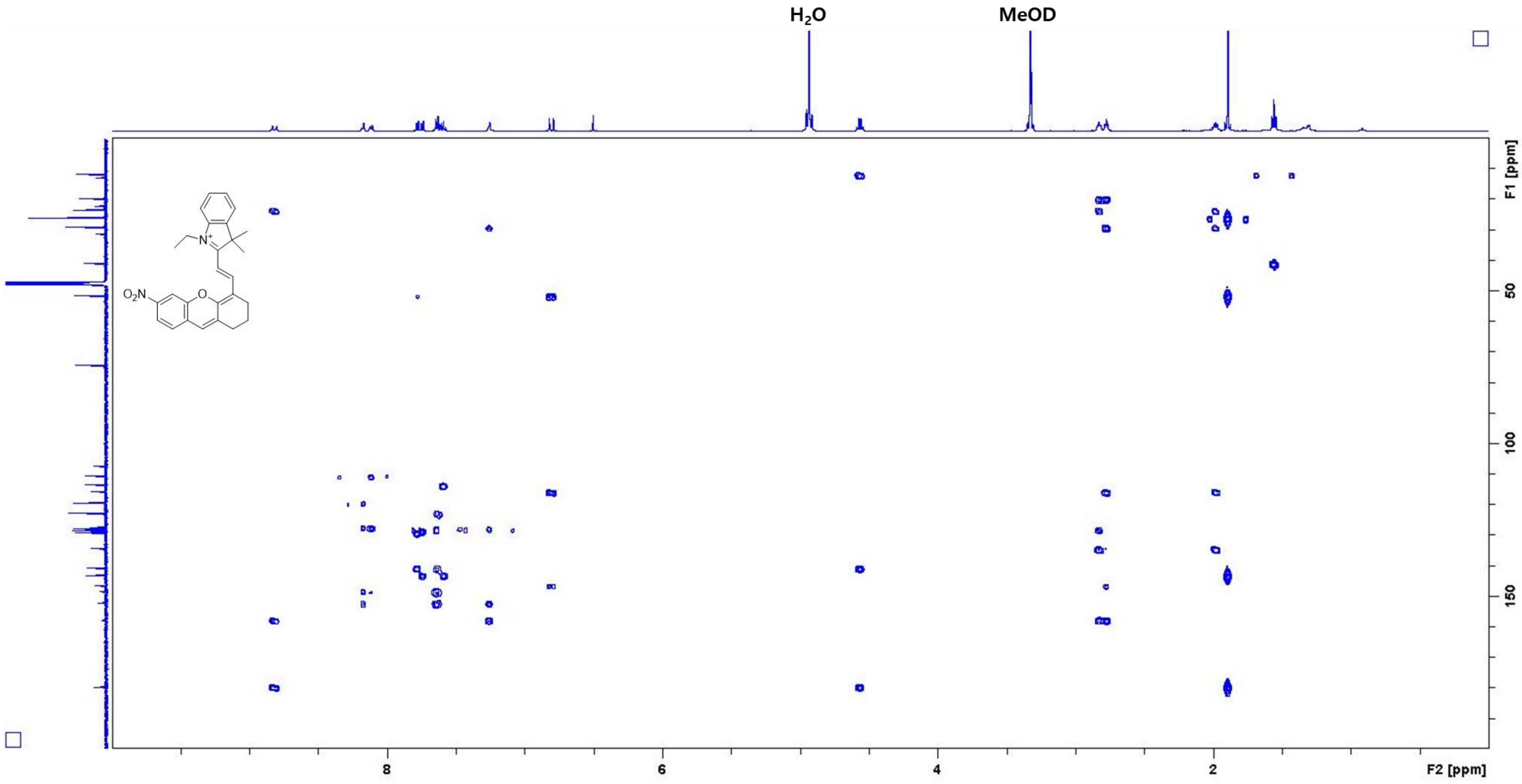

Figure S20. HMBC spectrum of compound **NIR-HCy-NO2 1** (500 MHz).

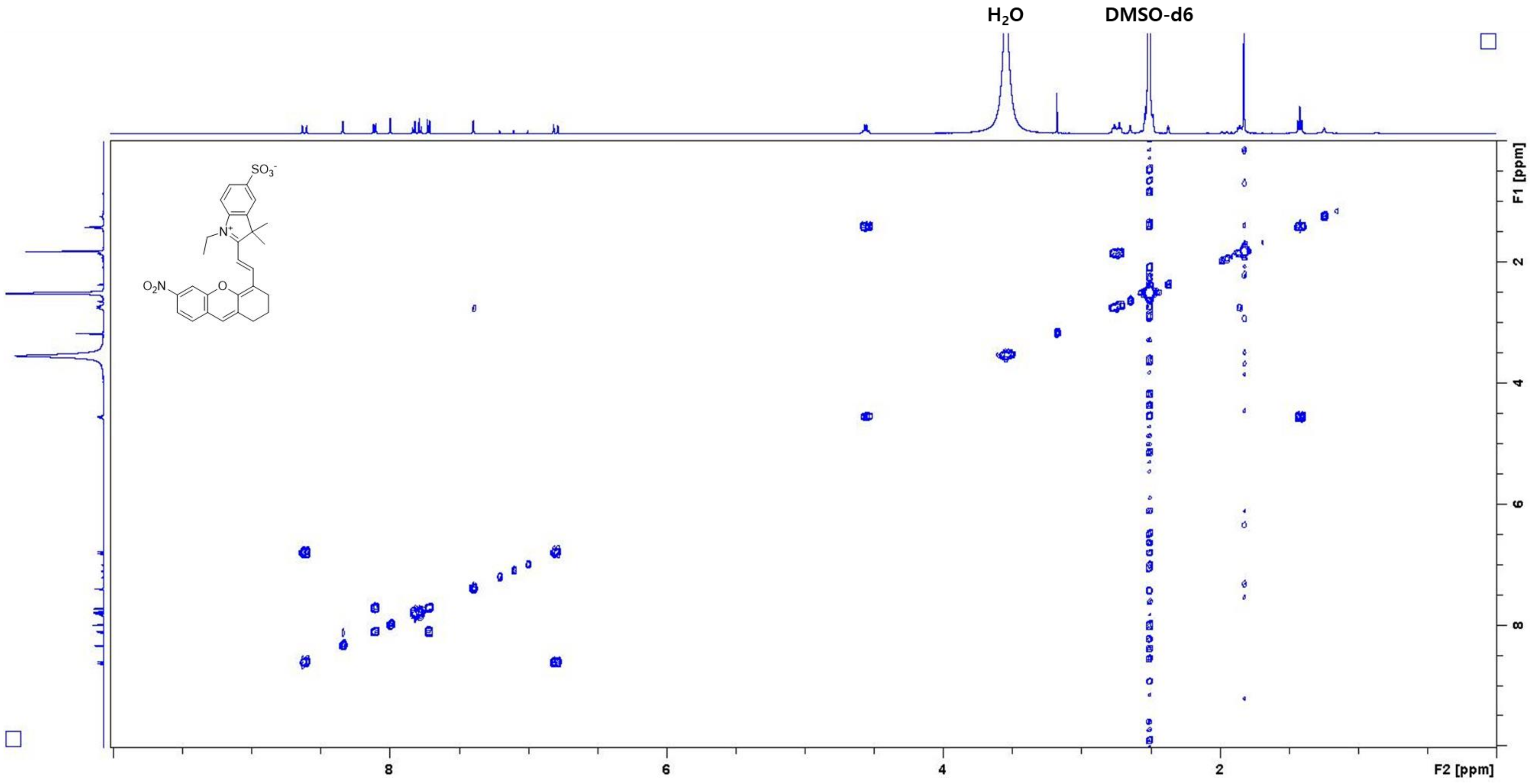

Figure S21. COSY spectrum of compound **NIR-HCy-NO₂ 2** (500 MHz).

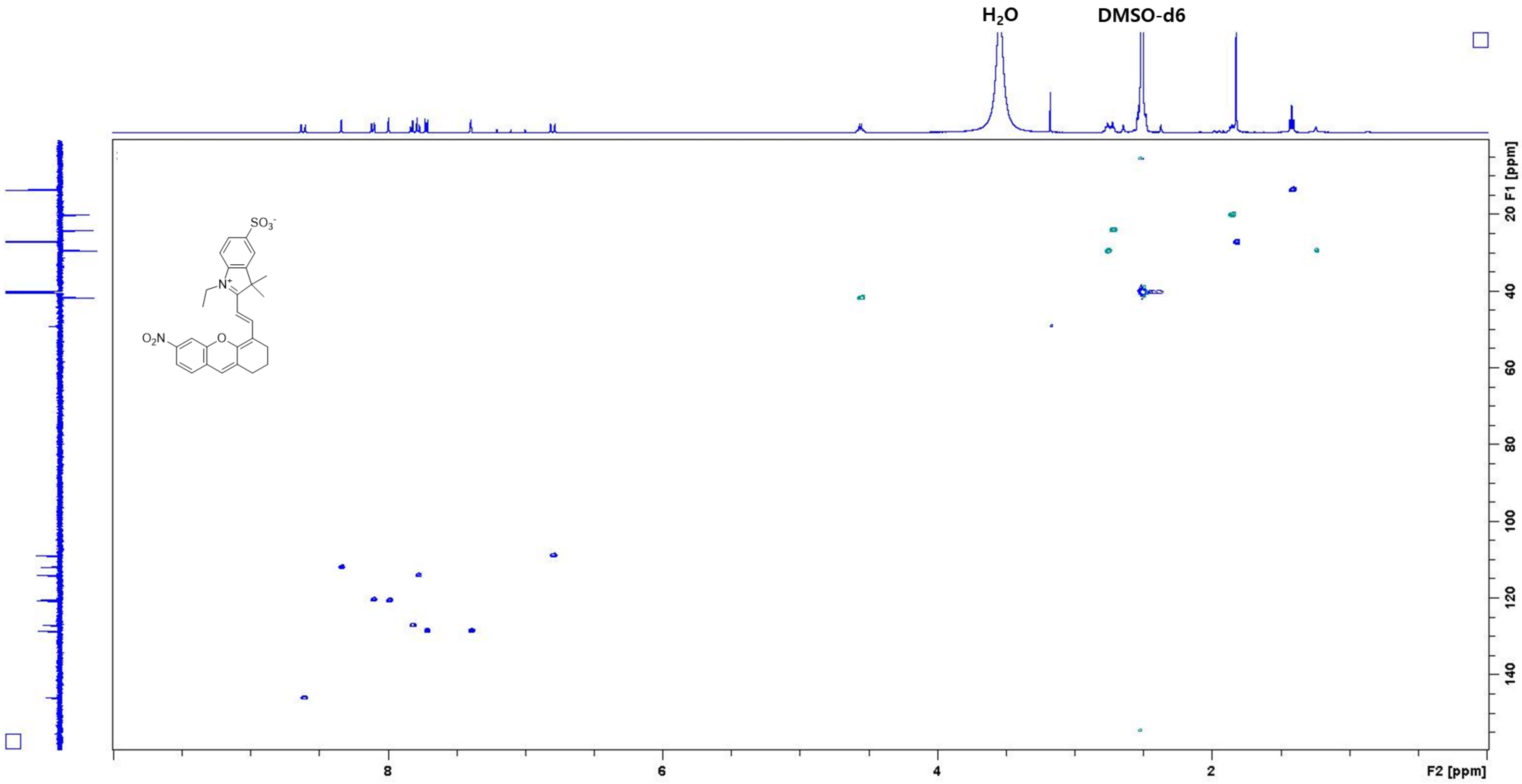

Figure S22. HSQC spectrum of compound **NIR-HCy-NO₂ 2** (500 MHz).

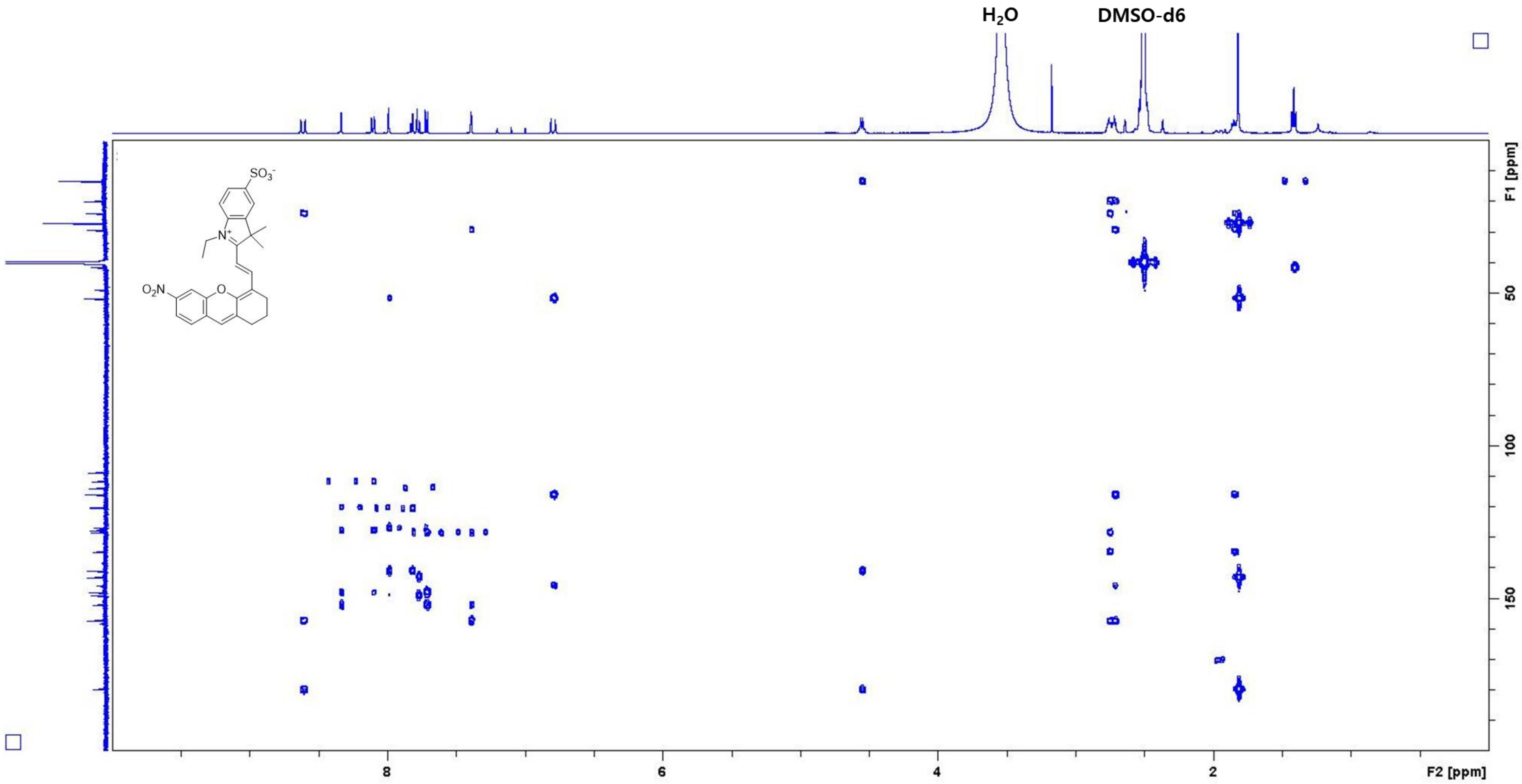

Figure S23. HMBC spectrum of compound **NIR-HCy-NO₂ 2** (500 MHz).

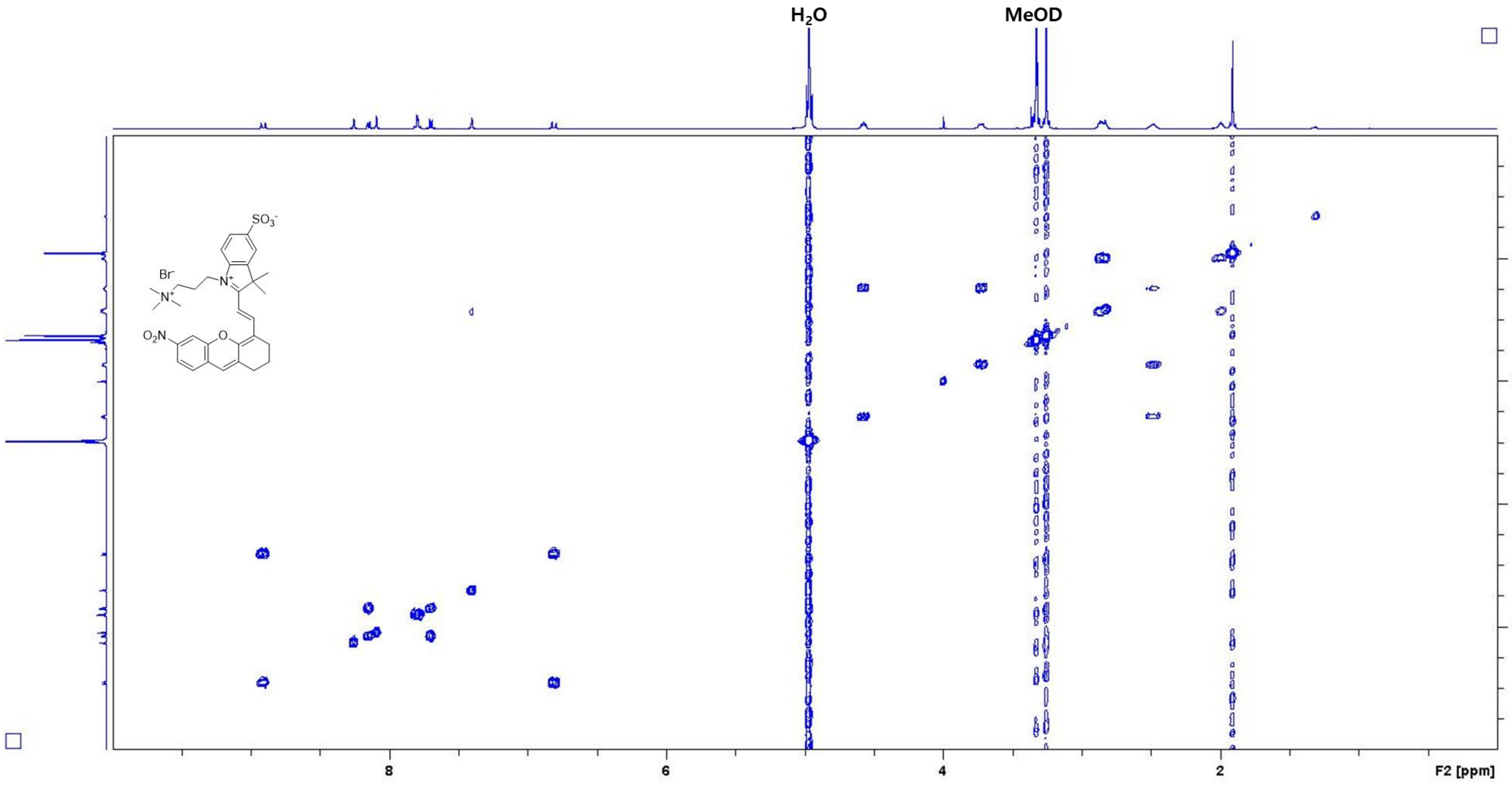

Figure S24. COSY spectrum of compound **NIR-HCy-NO$_2$ 3** (500 MHz).

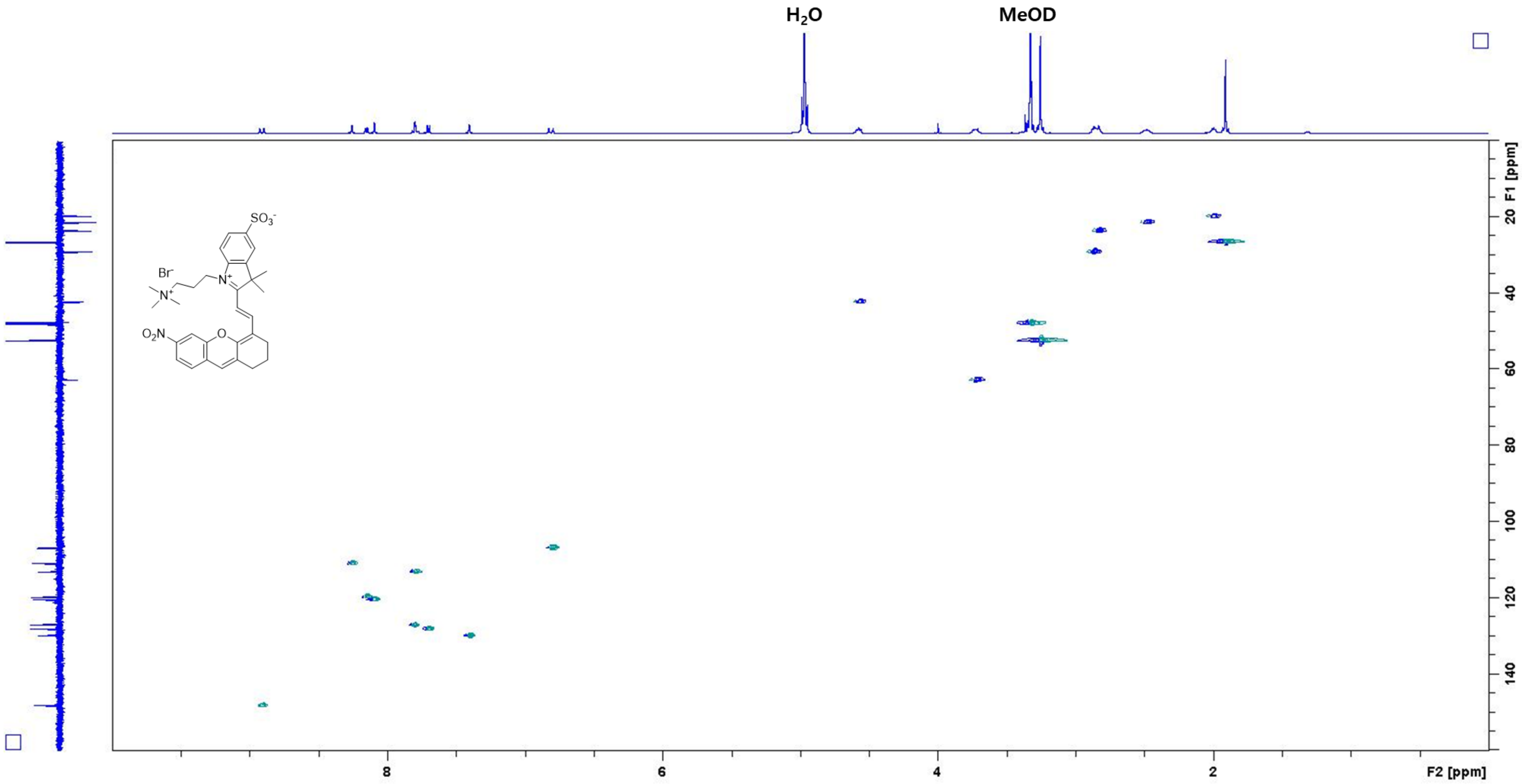

Figure S25. HSQC spectrum of compound **NIR-HCy-NO$_2$ 3** (500 MHz).

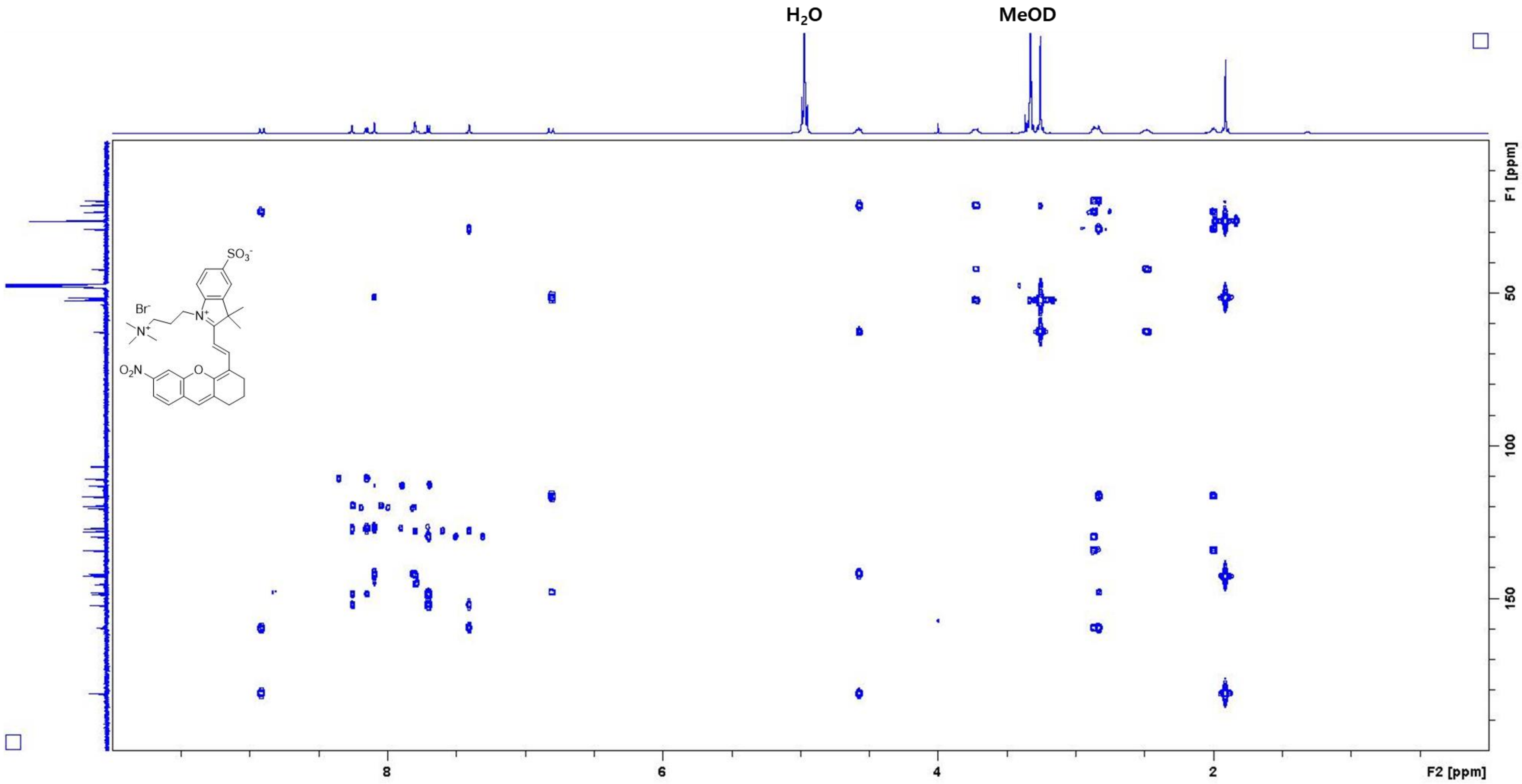

Figure S26. HMBC spectrum of compound **NIR-HCy-NO₂ 3** (500 MHz).

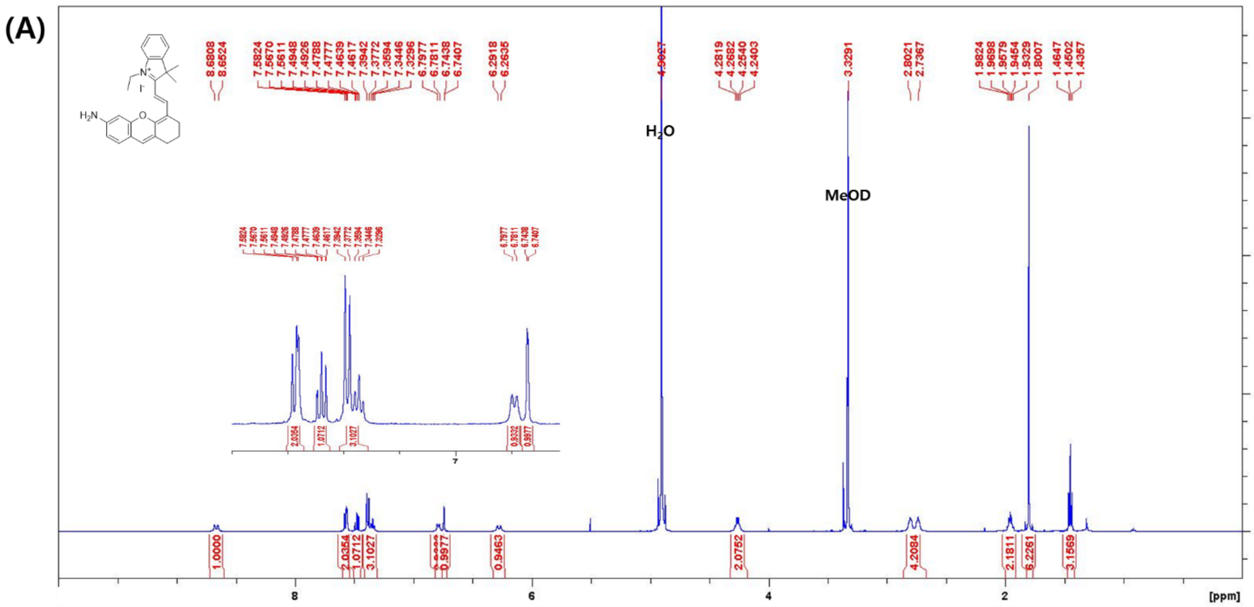

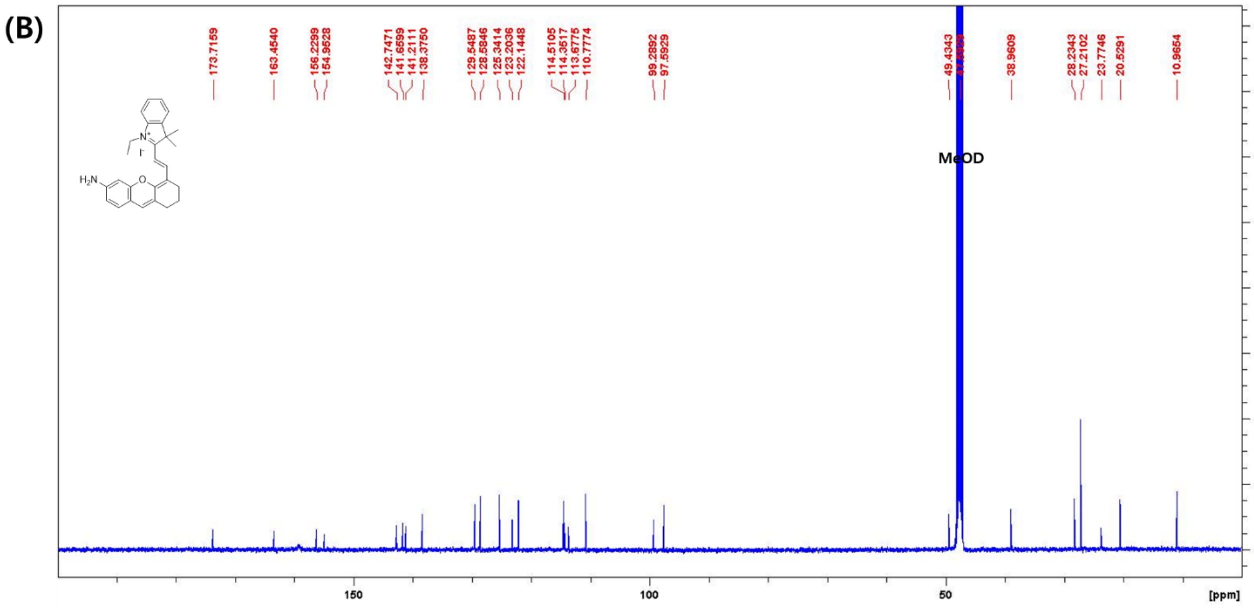

Figure S27. ^{1}H (A) and ^{13}C (B) NMR spectrum of compound **NIR-HCy-NH$_2$ 1**.

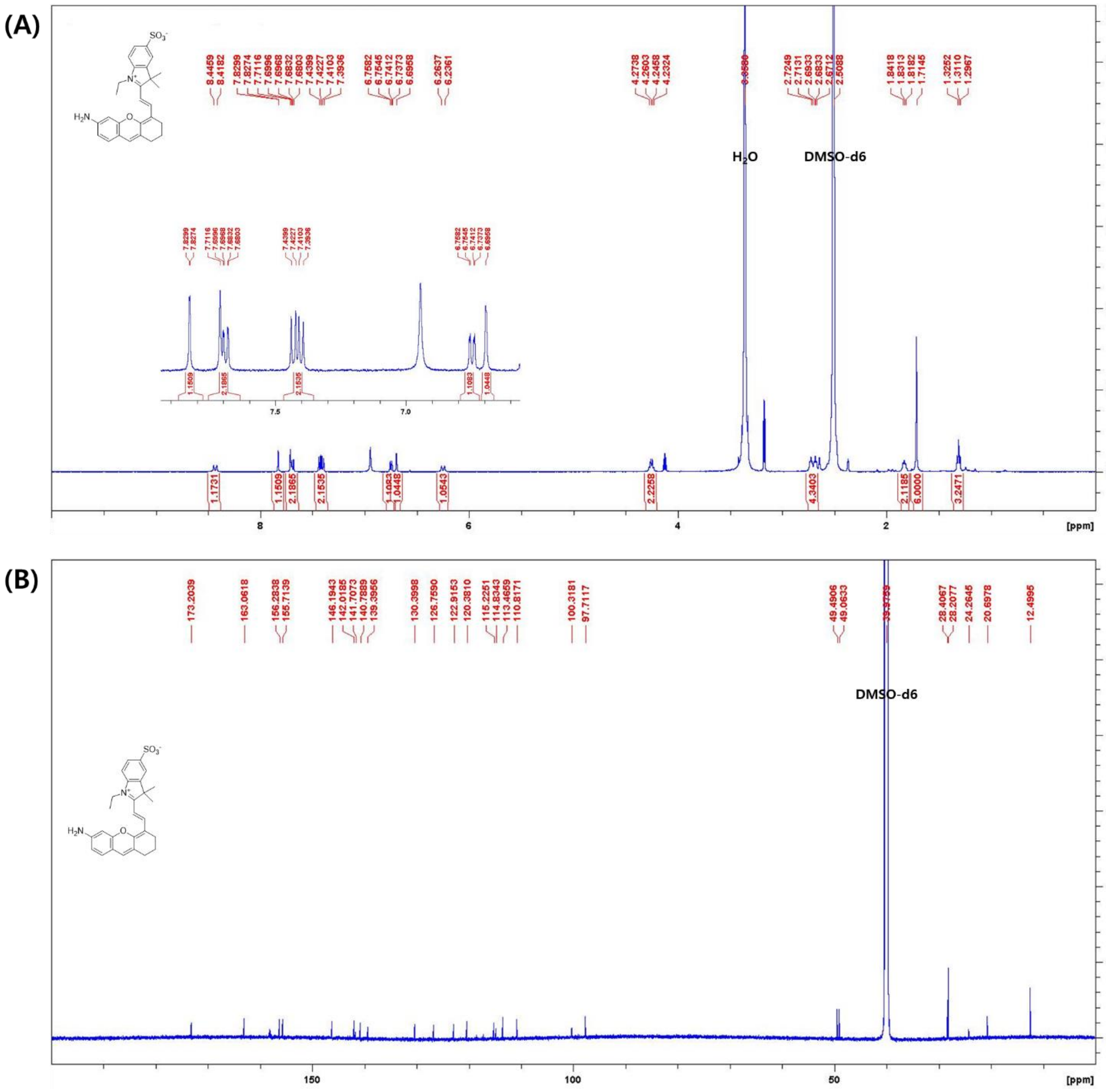

Figure S28. ^1H (A) and ^{13}C (B) NMR spectrum of compound **NIR-HCy-NH$_2$ 2**.

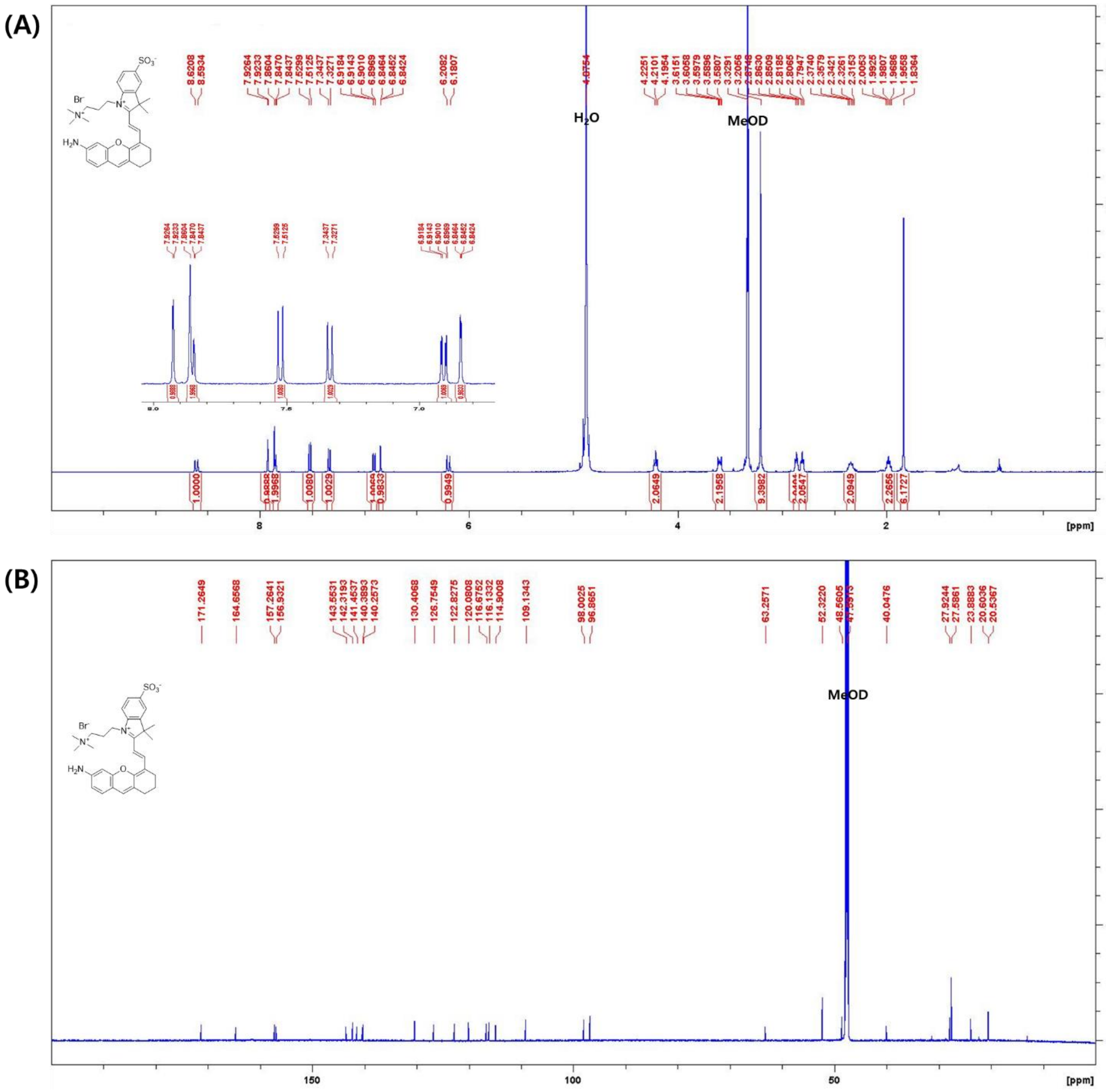

Figure S29. ^{1}H (A) and ^{13}C (B) NMR spectrum of compound **NIR-HCy-NH$_2$ 3**.

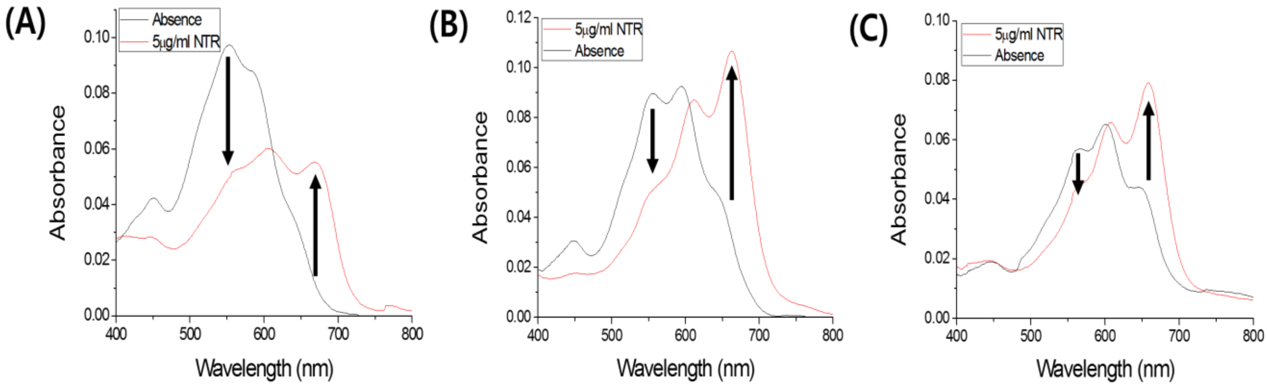

Figure S30. UV-Visible spectra of **NIR-HCy-NO₂ 1-3** (5 µM) reacted with (red line) and without (black line) 5 µg/mL NTR and 50 µM NADH for 30 minutes at 37°C. (A) **NIR-HCy-NO₂ 1** in 1X PBS (pH 7.4, 20% (v/v) ACN), (B) **NIR-HCy-NO₂ 2** in 1X PBS (pH 7.4, 5% (v/v) ACN), (C) **NIR-HCy-NO₂ 3** in 1X PBS (pH 7.4).

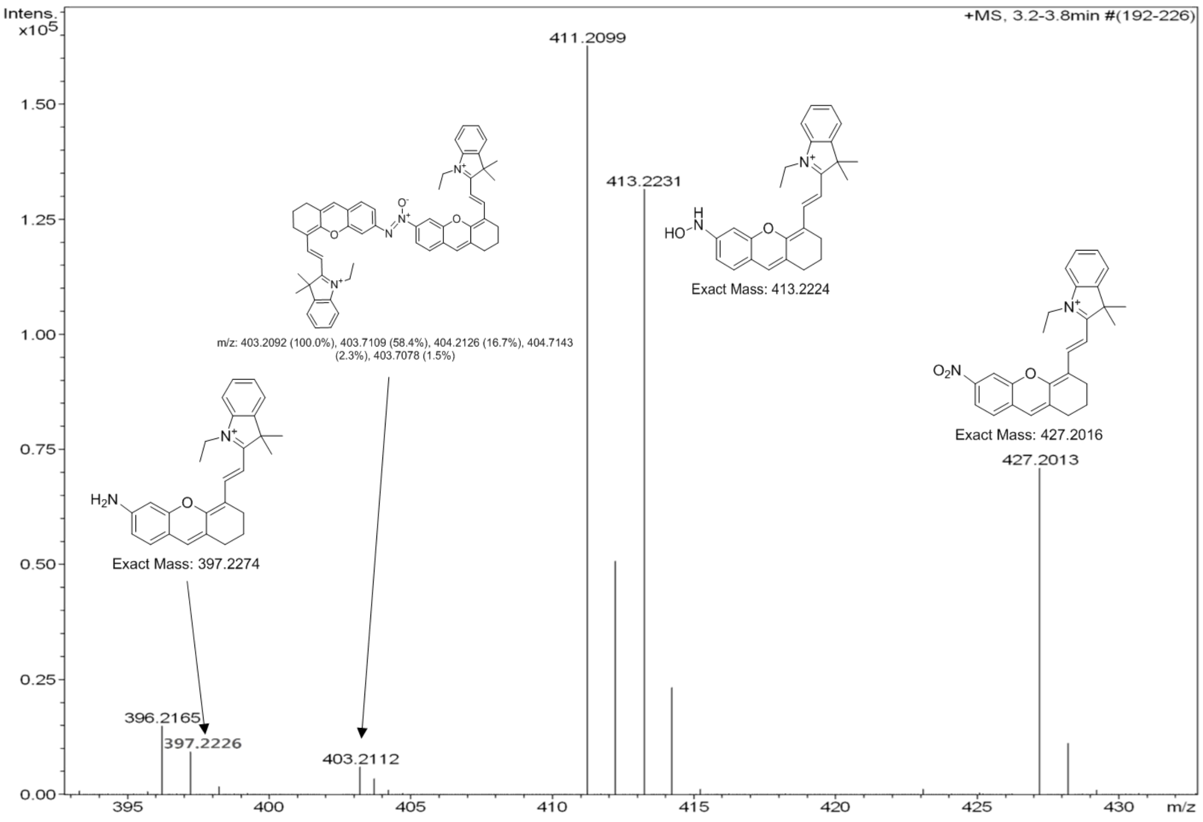

Figure S31. Mass spectrum of **NIR-HCy-NO₂ 1** and intermediates and product of reduced **NIR-HCy-NO₂ 1**.

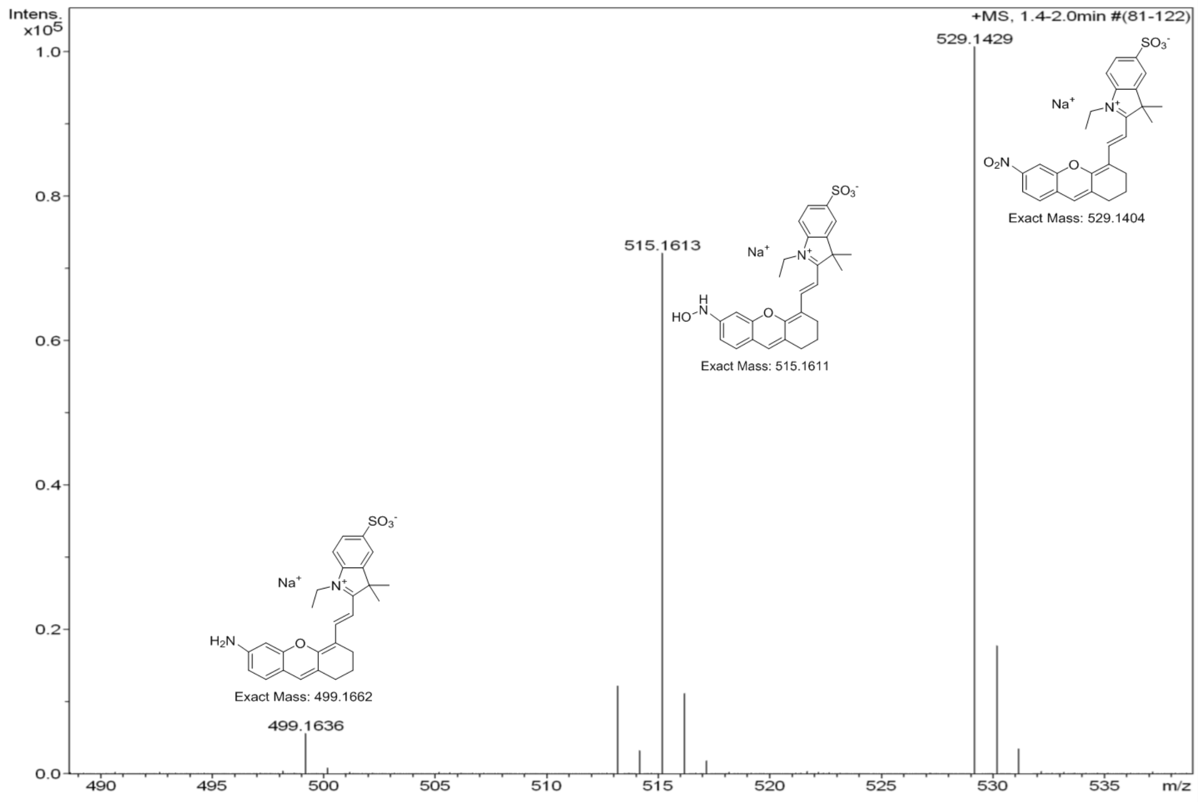

Figure S32. Mass spectrum of **NIR-HCy-NO₂ 2** and intermediate and product of reduced **NIR-HCy-NO₂ 2**.

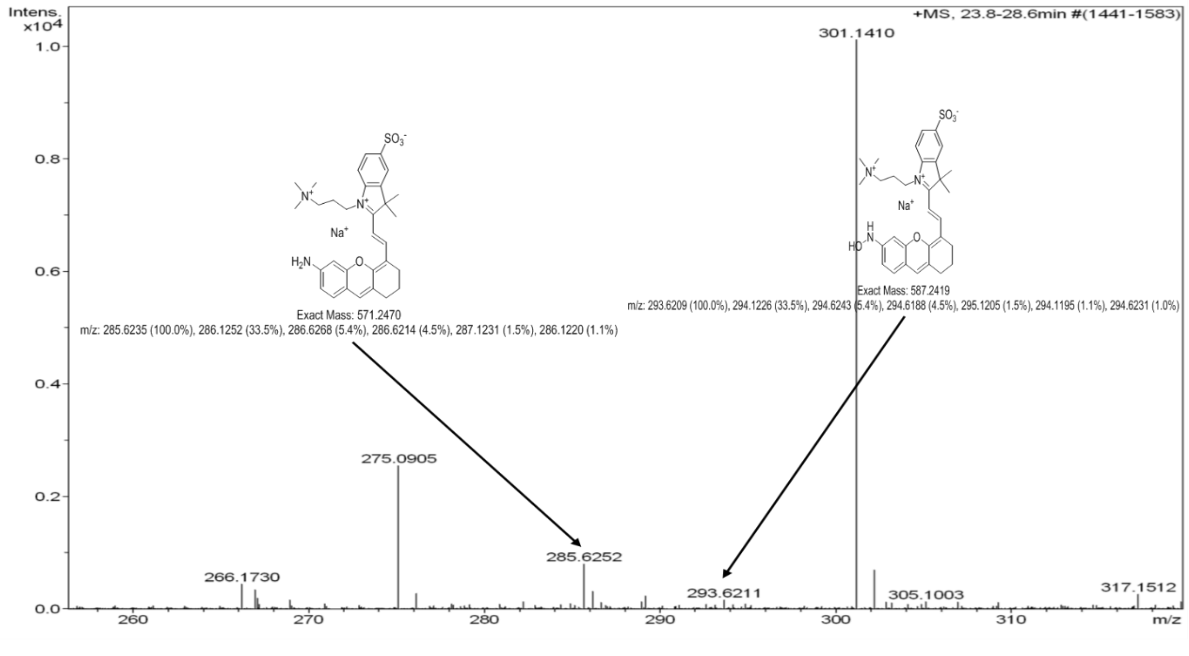

Figure S33. Mass spectrum of **NIR-HCy-NO₂ 3** and product of reduced **NIR-HCy-NO₂ 3**.

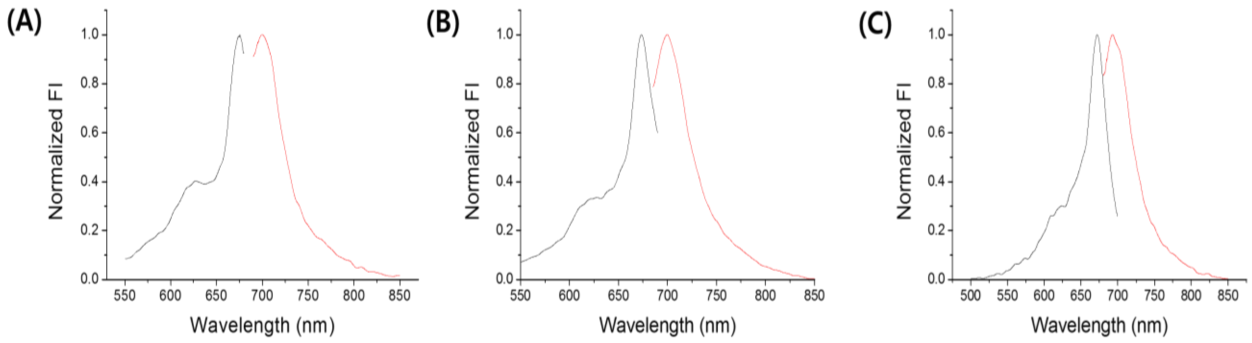

Figure S34. Excitation (black line) and emission (red line) fluorescence spectra of **NIR-HCy-NO$_2$ 1-3** (5 μM) reacted with 5 μg/mL NTR in the presence of 50 μM NADH for 30 minutes at 37°C. (A) **NIR-HCy-NO$_2$ 1** in 1X PBS (pH 7.4, 20% (v/v) ACN), (B) **NIR-HCy-NO$_2$ 2** in 1X PBS (pH 7.4, 5% (v/v) ACN), (C) **NIR-HCy-NO$_2$ 3** in 1X PBS (pH 7.4). Where normalized FI is the fluorescence intensity that is divided by the peak value in the fluorescence spectrum. The emission spectra were recorded using λ_{ex} = 672 nm.

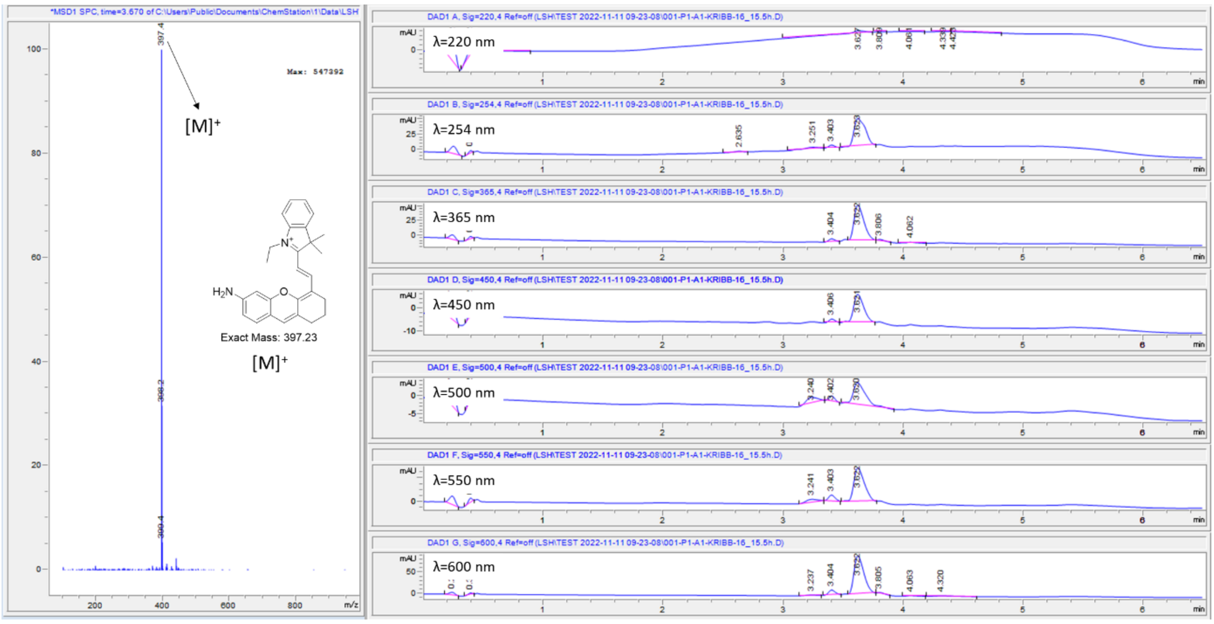

Figure S35. LC-MS of **NIR-HCy-NH$_2$ 1**.

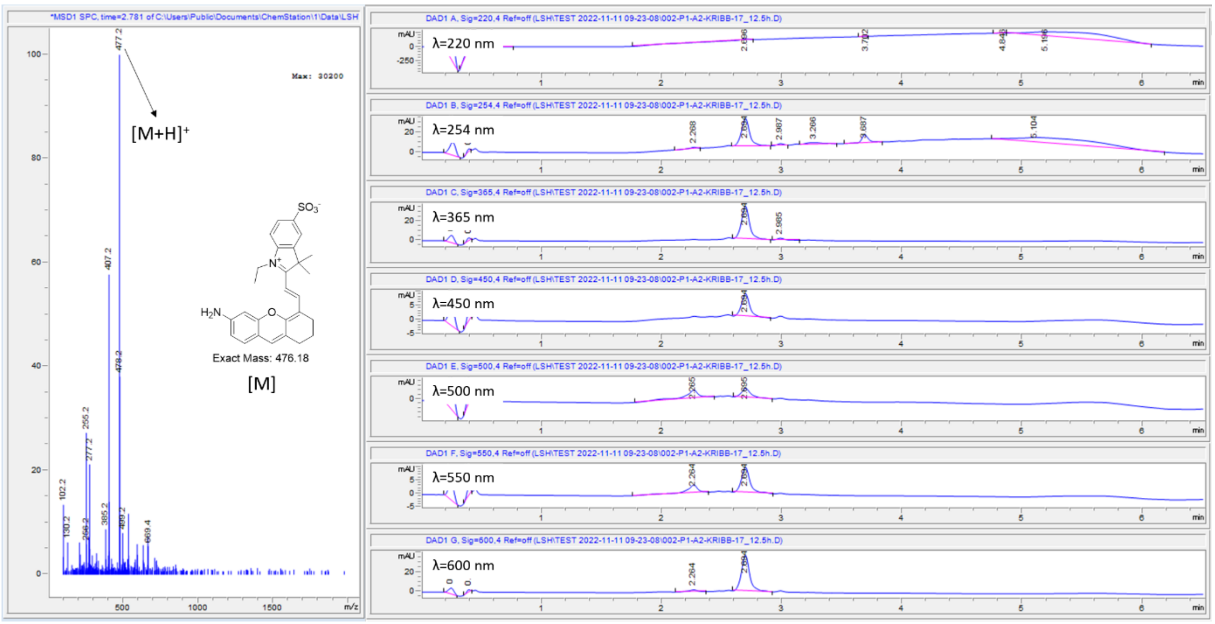

Figure S36. LC-MS of **NIR-HCy-NH$_2$ 2**.

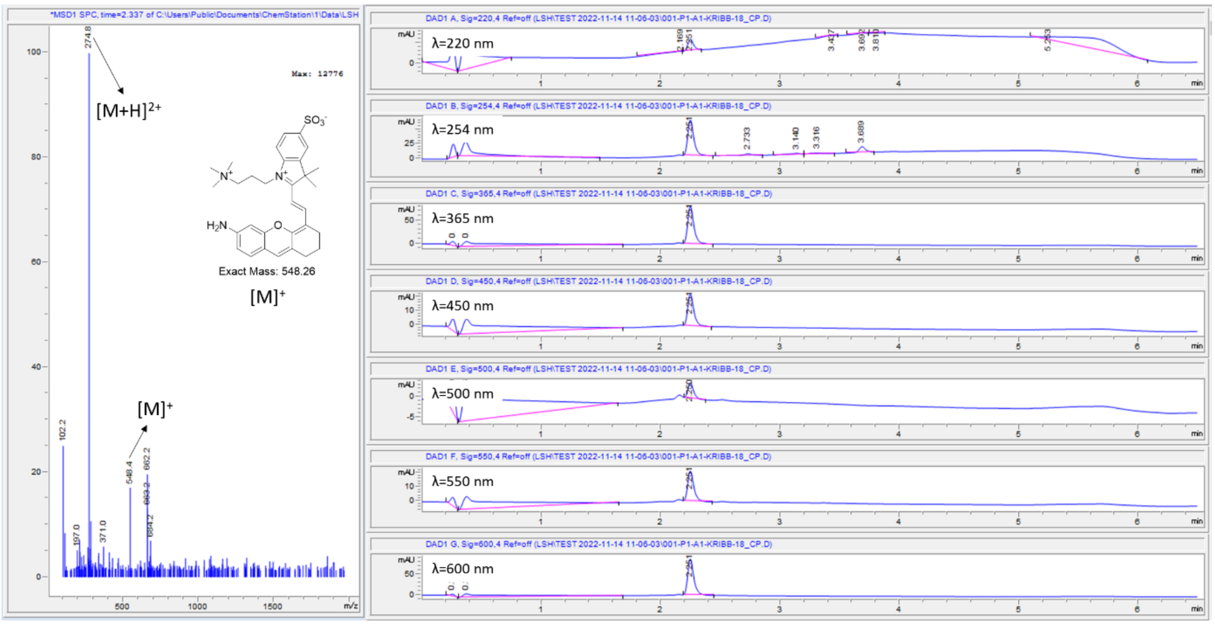

Figure S37. LC-MS of **NIR-HCy-NH$_2$ 3**.

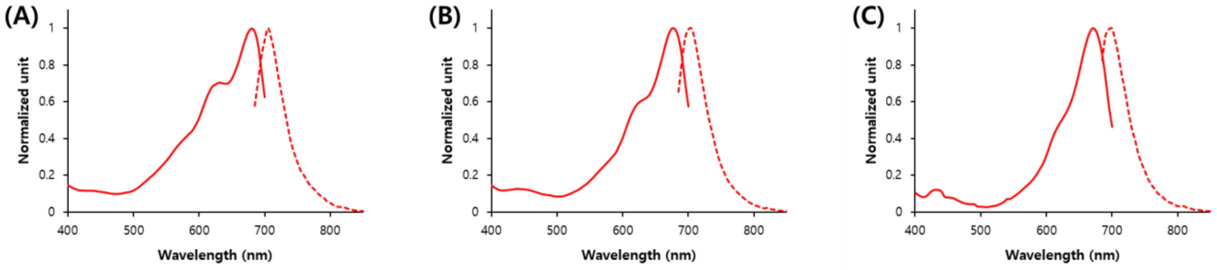

Figure S38. Absorbance and emission spectra of **NIR-HCy-NH₂ 1-3**. (A) **NIR-HCy-NH₂ 1** in in 1X PBS (pH 7.4, 20% (v/v) ACN), (B) **NIR-HCy-NH₂ 2** in 1X PBS (pH 7.4, 5% (v/v) ACN), (C) **NIR-HCy-NH₂ 3** in 1X PBS (pH 7.4). The emission spectra were recorded using λ_{ex} = 672 nm.

Table S1. Optical properties of **NIR-HCy-NO₂** and **NIR-HCy-NH₂**.

Compound	λ_{abs} (nm)	λ_{em} (nm)	ε ($M^{-1}cm^{-1}$)	Φ_{fl}	Solvent
**NIR-HCy-NO₂ 1**	552	-	4.17 x 10^4	0.0005	10mM PBS (pH 7.4, 20% ACN)
**NIR-HCy-NO₂ 2**	594	-	3.57 x 10^4	0.0003	10mM PBS (pH 7.4, 5% ACN)
**NIR-HCy-NO₂ 3**	600	-	3.59 x 10^4	0.0004	10mM PBS (pH 7.4)
**NIR-HCy-NH₂ 1**	680	706	8.64 x 10^4	0.013	10mM PBS (pH 7.4, 20% ACN)
**NIR-HCy-NH₂ 2**	677	703	1.07 x 10^5	0.021	10mM PBS (pH 7.4, 5% ACN)
**NIR-HCy-NH₂ 3**	671	696	6.56 x 10^4	0.025	10mM PBS (pH 7.4)

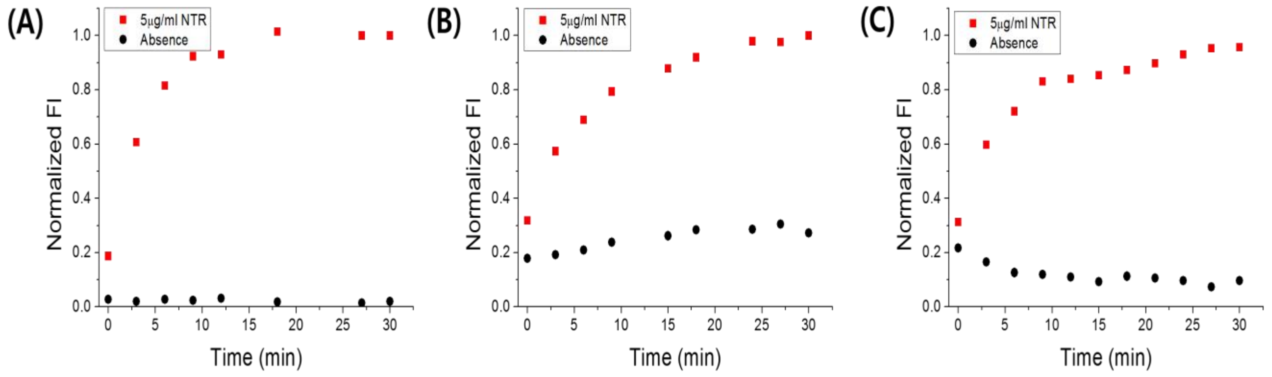

Figure S39. Time course of the reaction of **NIR-HCy-NO₂ 1-3** in the presence of 5 μg/mL NTR and 50 μM NADH over 30 minutes. (A) **NIR-HCy-NO₂ 1** in 1X PBS (pH 7.4, 20% (v/v) ACN), (B) **NIR-HCy-NO₂ 2** in 1X PBS (pH 7.4, 5% (v/v) ACN), (C) **NIR-HCy-NO₂ 3** in 1X PBS (pH 7.4). Where normalized FI is the fluorescence intensity that is divided by the peak value in the fluorescence spectrum.

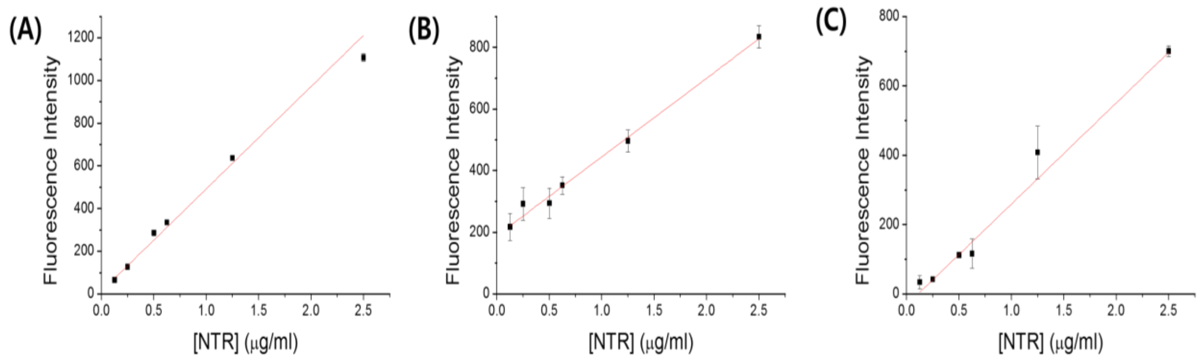

Figure S40. Fluorescence intensities of **NIR-HCy-NO2** (5 μM) reacted with various concentration of NTR (0.125-5 μg/mL) in the presence of 50 μM NADH for 30 minutes at 37°C. (A) **NIR-HCy-NO2 1** reacted in 1X PBS (pH 7.4, 20% (v/v) ACN), (B) **NIR-HCy-NO2 2** in 1X PBS (pH 7.4, 5% (v/v) ACN), (C) **NIR-HCy-NO2 3** in 1X PBS (pH 7.4).

Table S2. Michaelis-Menten kinetic parameters of **NIR-HCy-NO2** and other substrates.

Substrate	V_{max} (μM/sec)	K_M (μM)	k_{cat} (sec^{-1})	Catalytic efficiency ($μM^{-1}·sec^{-1}$)	Ref.
NIR-HCy-NO2 1	0.56 ± 0.004	24.67 ± 3.99	5.33±0.04	0.22 ± 0.03	This work
NIR-HCy-NO2 2	0.20 ± 0.005	14.69 ± 0.84	1.88±0.05	0.13 ± 0.005	This work
NIR-HCy-NO2 3	0.13 ± 0.008	23.02 ± 8.12	1.22±0.08	0.05 ± 0.05	This work
Nitrofurazone	-	160 ± 6	10.7 ± 0.1	0.15 ± 0.02	*J. Biol. Chem.* **280**, 13256-64 (2005)
Probe 1	3.7	32.2	-	-	*Biosens. Bioelectron.* **63**, 112-6 (2015)

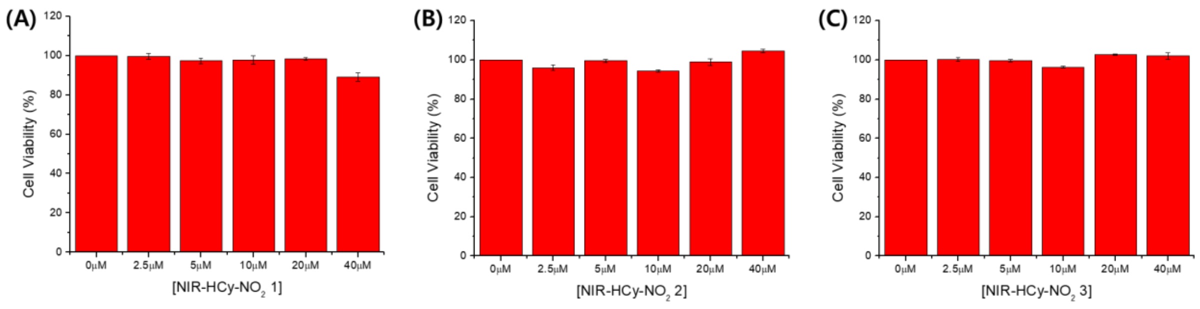

Figure S41. Cell Cytotoxicity test of **NIR-HCy-NO2 1-3**. Cell viability of A549 treated with various concentration of **NIR-HCy-NO2 1-3** for 1 hour. (A) **NIR-HCy-NO2 1** in DMEM (10% FBS, 1% P/S), (B) **NIR-HCy-NO2 2** in DMEM (10% FBS, 1% P/S), (C) **NIR-HCy-NO2 3** in DMEM (10% FBS, 1% P/S).

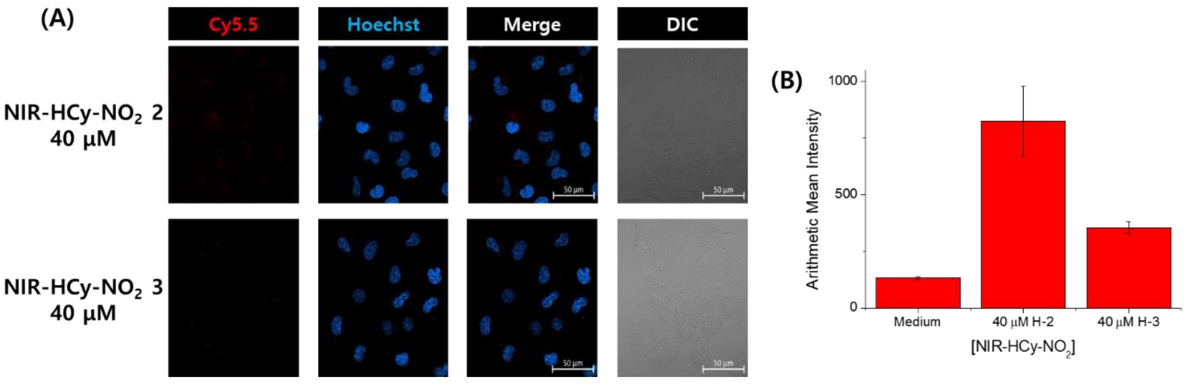

Figure S42. Confocal fluorescence images of **NIR-HCy-NO₂ 2** and **NIR-HCy-NO₂ 3** in living A549 cells and the arithmetic mean intensity of **NIR-HCy-NO₂ 2** and **NIR-HCy-NO₂ 3** obtained from (A). (A) Intracellular NTR activity fluorescence and bright images of **NIR-HCy-NO₂ 2** and **NIR-HCy-NO₂ 3** in A549 cells. (B) Arithmetic mean intensity bar graph of medium and (A). The cell experiments were performed under normoxia condition.

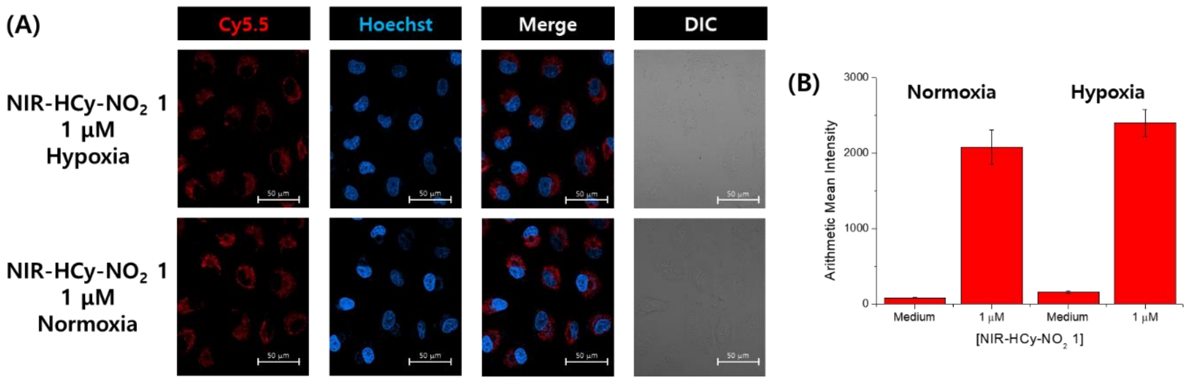

Figure S43. Confocal fluorescence images comparison of **NIR-HCy-NO₂ 1** between normoxia and hypoxia and the arithmetic mean intensity of **NIR-HCy-NO₂ 1** obtained from (A). (A) Intracellular NTR activity fluorescence and bright images of 1 µM **NIR-HCy-NO₂ 1** in A549 cells under normoxic and hypoxic condition. (B) Arithmetic mean intensity bar graph of medium and (A).

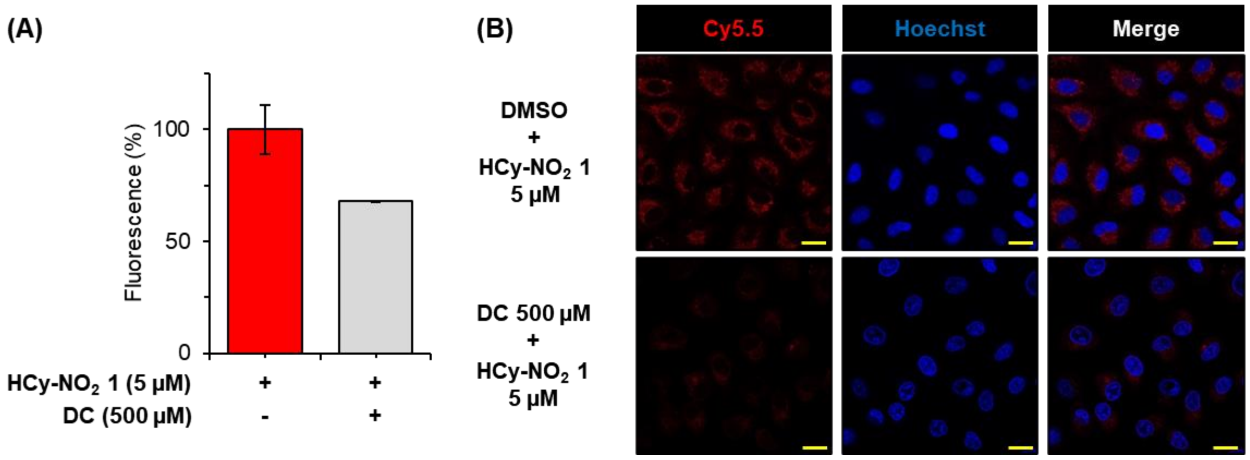

Figure S44. Effect of reductase inhibitor dicoumarol on **NIR-HCy-NO₂ 1**-mediated fluorescence enhancement in A549 cells. (A) A549 cells were treated **NIR-HCy-NO₂ 1** (5 µM) for 20 min in the presence or absence of dicoumarol (DC, 500 µM) for 4 hours, and then the fluorescence intensity was measured. (B) Confocal fluorescence images of A549 cells stained with **NIR-HCy-NO₂ 1** (5 µM) in the presence or absence of DC (500 µM). The cell experiments were performed under normoxia condition. The scale bar indicates 20 µm.

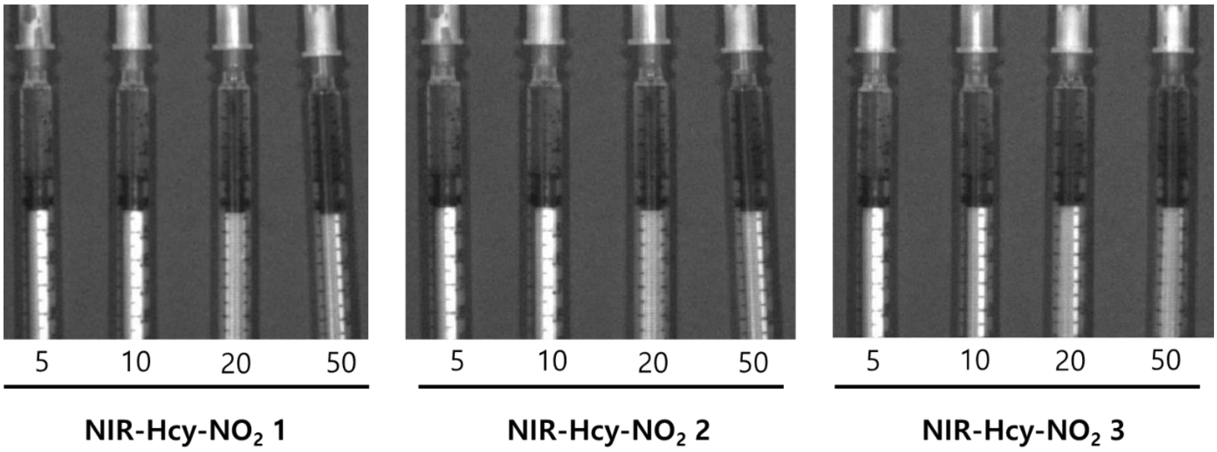

Figure S45. IVIS images of 5-50 μM **NIR-HCy-NO$_2$ 1-3** in syringe.

Reference

1) Kubin, R.F.; Fletcher, A. N. *J. Lumin.* **1982**, *27*, 455-462.

2) Choi, H. S.; Nasr, K.; Alyabyev, S.; Feith, D.; Lee, J. H.; Kim, S. H.; Ashitate, Y.; Hyun, H.; Patonay, G.; Strekowski, L.; Henary, M.; Frangioni, J. V. *Angew. Chem. Int. Ed.* **2011**, *50*, 6258-63.
